# Analysis of cardiac single-cell RNA-sequencing data can be improved by the use of artificial-intelligence-based tools

**DOI:** 10.1038/s41598-023-32293-1

**Published:** 2023-04-26

**Authors:** Thanh Nguyen, Yuhua Wei, Yuji Nakada, Jake Y. Chen, Yang Zhou, Gregory Walcott, Jianyi Zhang

**Affiliations:** 1 Department of Biomedical Engineering, University of Alabama at Birmingham, Birmingham, AL 35233 USA; 2 Department of Medicine, Cardiovascular Diseases, University of Alabama at Birmingham, Birmingham, AL 35233 USA; 3 Informatics Institute, School of Medicine, University of Alabama at Birmingham, Birmingham, AL 35233 USA; 4 Department of Biomedical Engineering, School of Medicine and School of Engineering, University of Alabama at Birmingham, 1670 University Blvd, Volker Hall G094J, Birmingham, AL 35233 USA

**Keywords:** Biomedical engineering, Cardiac regeneration, Machine learning

## Abstract

Single-cell RNA sequencing (scRNAseq) enables researchers to identify and characterize populations and subpopulations of different cell types in hearts recovering from myocardial infarction (MI) by characterizing the transcriptomes in thousands of individual cells. However, the effectiveness of the currently available tools for processing and interpreting these immense datasets is limited. We incorporated three Artificial Intelligence (AI) techniques into a toolkit for evaluating scRNAseq data: AI Autoencoding separates data from different cell types and subpopulations of cell types (cluster analysis); AI Sparse Modeling identifies genes and signaling mechanisms that are differentially activated between subpopulations (pathway/gene set enrichment analysis), and AI Semisupervised Learning tracks the transformation of cells from one subpopulation into another (trajectory analysis). Autoencoding was often used in data denoising; yet, in our pipeline, Autoencoding was exclusively used for cell embedding and clustering. The performance of our AI scRNAseq toolkit and other highly cited non-AI tools was evaluated with three scRNAseq datasets obtained from the Gene Expression Omnibus database. Autoencoder was the only tool to identify differences between the cardiomyocyte subpopulations found in mice that underwent MI or sham-MI surgery on postnatal day (P) 1. Statistically significant differences between cardiomyocytes from P1-MI mice and mice that underwent MI on P8 were identified for six cell-cycle phases and five signaling pathways when the data were analyzed via Sparse Modeling, compared to just one cell-cycle phase and one pathway when the data were analyzed with non-AI techniques. Only Semisupervised Learning detected trajectories between the predominant cardiomyocyte clusters in hearts collected on P28 from pigs that underwent apical resection (AR) on P1, and on P30 from pigs that underwent AR on P1 and MI on P28. In another dataset, the pig scRNAseq data were collected after the injection of CCND2-overexpression Human-induced Pluripotent Stem Cell-derived cardiomyocytes (^CCND2^hiPSC) into injured P28 pig heart; only the AI-based technique could demonstrate that the host cardiomyocytes increase proliferating by through the HIPPO/YAP and MAPK signaling pathways. For the cluster, pathway/gene set enrichment, and trajectory analysis of scRNAseq datasets generated from studies of myocardial regeneration in mice and pigs, our AI-based toolkit identified results that non-AI techniques did not discover. These different results were validated and were important in explaining myocardial regeneration.

## Introduction

Cardiomyocytes comprise most of the cardiac mass^1^ but are among the least proliferative cells in adult mammals^2^; thus, cardiac disease or injury frequently progresses to heart failure because the heart cannot regenerate damaged myocardial tissue^3^. However, cardiomyocytes are robustly proliferative during the fetal development^4–7^, and when myocardial infarction (MI) was induced on postnatal day (P) 1 in newborn piglets, the animals recovered completely by P30 with no decline in contractile performance and negligible myocardial scarring^6^. Furthermore, although this residual fetal/neonatal capacity for cardiomyocyte proliferation is normally lost by postnatal day 2–3 (P2-P3), cardiomyocytes in pigs that underwent apical resection surgery (AR) on P1 retained a latent capacity of the active cardiomyocyte cell cycle for at least four weeks afterward. They completely regenerated the myocardial tissue that was lost to secondary acute myocardial infarction to a left anterior descending coronary artery (LAD) occlusion on P28^8,9^. Collectively, these observations suggest that a thorough characterization of how cardiomyocyte gene expression changes in response to AR on P1 (AR_P1_), MI on P28 (MI_P28_), or both AR_P1_ and MI_P28_ could provide key insights into the mechanisms that govern cardiomyocyte proliferation and how they may be manipulated to improve recovery from myocardial disease^3,10–12^.

The heart is composed of numerous cell types, and individual cells within a single lineage likely respond differently to myocardial injury. High-throughput single-cell RNA sequencing (scRNAseq) can accommodate this heterogeneity by enabling researchers to characterize the transcriptomes for thousands of individual cells, but the dimensionality of the resulting dataset is immense. Furthermore, even in regenerating hearts that responded to myocardial injury, only a very small proportion of cardiomyocytes are proliferating at any given time point. In contrast, others are likely apoptotic or hypertrophic, and individual cardiomyocytes can transition from one subpopulation to another over time. Thus, adequate interpretation of an scRNAseq dataset^9,13–18^ requires the application of bioinformatics tools that can (1) separate data from different cell types and subpopulations of cell types (i.e., "cluster" analysis)^19^, (2) identify which genes and signaling mechanisms are differentially activated between subpopulations (i.e., "pathway/gene set enrichment" analysis)^20–23^, and (3) track the transformation of cells from one subpopulation into another (i.e., "trajectory" analysis)^24–27^.

When scRNAseq data from studies conducted in a pig cardiac double-injury (AR_P1_ followed by MI_P28_) model was processed with current state-of-the-art bioanalysis tools, ten distinct cardiomyocyte subpopulations were identified, one of which reverted to a more perinatal-like phenotype characterized by increases in cell-cycle activity and proliferation^9^; however, the regulatory molecules and signaling pathways responsible for activating cardiomyocyte proliferation could not be identified. Thus, we have constructed a bioinformatics toolkit incorporating several techniques from the field of Artificial Intelligence (AI)^28^ and then tested it with scRNAseq datasets obtained from the Gene Expression Omnibus (GEO) database and one obtained from the Human Heart Cell Atlas^17^. The results presented in this report suggest that our AI-based approach was more effective than other highly cited non-AI bioinformatics techniques for processing and interpreting scRNAseq data.

## Methods

### scRNAseq datasets

The scRNAseq analytic techniques were tested with datasets obtained from studies conducted in mouse (GEO dataset number GSE130699)^14^ and pig (GEO dataset number GSE185289)^9^ models of myocardial infarction (MI). Mice underwent MI induction surgery on P1 or P8 (P1-MI or P8-M1, respectively) or Sham surgery on P1 or P8 (P1-Sham or P8-Sham, respectively), and cardiac tissues were collected 1 (D1) or 3 (D3) days later (Table 1). Data were analyzed for a total of 31,586 cells, including cardiomyocytes, endothelial cells, fibroblasts, and immune cells. Pigs underwent AR_P1_, MI_P28_, both AR_P1_ and MI_P28_ (AR_P1_MI_P28_), or neither surgical procedure (CTL). Tissues were collected from the border zone of the infarcted MI_P28_ and AR_P1_MI_P28_ animals on P30, P35, P42, and P56, or from the corresponding region of hearts in AR_P1_ and CTL animals on P28 and P56. Tissues were also collected from CTL animals on P1 and from fetal pig hearts. Data were analyzed for a total of 250,700 pig cells, including cardiomyocytes, smooth muscle cells, endothelial cells, fibroblasts, skeletal muscle cells, and immune cells.

**Table 1:** Mouse scRNA dataset (GSE130699)^14^.

Sample ID	Postnatal day of MI or Sham surgery	Postnatal day of cardiac tissue collection	Proliferative capacity	Number of cells
P1-MI-D1	1	2	Strong	3209
P1-MI-D3	1	4	Strong	2694
P8-MI-D1	8	9	Weak	3801
P8-MI-D3	8	11	Weak	4795
P1-Sham-D1	1	2	Moderate	2825
P1-Sham-D3	1	4	Weak	5740
P8-Sham-D1	8	9	Weak	4568
P8-Sham-D3	8	11	Weak	3954

Third, we downloaded the scRNAseq data from the Heat Cell Atlas^17^ (https://www.heartcellatlas.org/), which is publicly available at the European Nucleotide Archieve accession number PRJEB39602. The dataset contained 486,134 cells, was divided into 154 samples, and was collected from 14 donors with unremarkable cardiovascular history^17^. For each donor, cells from the left ventricle, right ventricle, left atrial, right atrial, and apex regions were obtained. There are five cell lineages in the dataset: cardiomyocyte (ventricular and atrial cardiomyocyte), vascular compartment (endothelial cell, smooth muscle cell, and pericyte), immune cells (monocyte-macrophage and lymphocyte), fibroblast, and neuronal (also called glial) cell.

Forth, in our previous work^29^, after ^CCND2^hiPSC were injected into the MI injury model on postnatal day P28, the pigs' cardiomyocytes increased proliferation. This was confirmed by counting the proportion of cardiomyocytes expressing cytokinesis-exclusive marker Aurora Kinase B (AURKB). In this work, we repeated the same experiment on two ischemic reperfusions (IR) on P28 pigs (IR_P28_). One pig was sacrificed one week, and the other was sacrificed four weeks after the ^CCND2^hiPSC injection. Also, we collected scRNAseq data in four pigs that underwent IR_P28_ injury without treatment as a control group. The total number of cells in this ^CCND2^hiPSC transplantation data is 34,451.

### Computer hardware

Data analysis was performed on an in-house DELL Precision 5820 Tower workstation computer equipped with an Intel^®^ Core™ i9-10920X 12-core CPU, 256 GB of memory, an Nvidia Quadro RTX4000 8 GB GPU, a 12-TB hard drive, and the most recent (as of November 2021) versions of all software programs, including Anaconda version 3, R version 4.1.2, Python version 3.8, and Matlab version 2021b.

### scRNAseq data integration and normalization

Data preprocessing, integration, and normalization were completed via Seurat^30^. Cells with fewer than 200 genes, fewer than 500 unique molecular identifiers (UMIs), more than 30,000 UMIs, or > 25% mitochondrial UMIs were omitted; the cutoff for mitochondrial genes was greater than the default setting (5%)^31^ because cardiomyocytes have an exceptionally high energy demand^32^. Total expression was multiplied by a factor of 10,000 and log-transformed (base 2), and variations in the number of genes and UMI detected per cell were scaled via the ScaleData function^31^ with vars.to.regress set to nUMI and nGenes. Normalization returned two gene-cell matrices: one in log scale, and the other the adjusted gene-cell count.

### Selection of non-AI techniques for comparative analyses

Non-AI tools for scRNAseq clustering (Table 2), pathway/gene set enrichment (Table 3), and trajectory analysis (Table 4) were selected from the online scRNA-Tools catalog (https://www.scrna-tools.org/tools), which tracked the use of 1027 scRNA-seq tools as of August 2021^33^. Clustering tools were filtered with the "Clustering" tag, pathway/gene set tools were filtered with the "Gene Sets" tag, and trajectory analysis tools were filtered with the "Ordering" tag. Then the filtering tools were ranked from the highest to the lowest number of citations. The five (clustering), three (pathway/geneset), and two (trajectory analysis) most highly cited tools that could be successfully installed on our workstation and did not produce technical errors when processing 31,586 mouse and 250,700 pig cells were chosen for comparative analyses. scRNAseq data were normalized via Seurat^30^ before clustering, and for tools that did not include an embedding step (RaceID, SC3, and CIDR), embedding was also performed via Seurat. Pathway/gene set and trajectory tools were implemented in Monocle combination with Seurat^30^, and the lists of differentially expressed genes were analyzed with the DAVID functional annotation tool^34^ to determine which pathways and gene sets were upregulated.

**Table 2:** Non-AI scRNAseq clustering tools.

Technique name	Version	Software platforms	# citations	Tutorial website
Seurat^30^	4.0	R	10,670	https://satijalab.org/seurat/articles/pbmc3k_tutorial.html
Scanpy^75^		Python	1579	https://scanpy-tutorials.readthedocs.io/en/latest/pbmc3k.html
RaceID^98^	3.0	R	1127	https://cran.r-project.org/web/packages/RaceID/vignettes/RaceID.html
SC3^99^		R	743	http://bioconductor.org/packages/release/bioc/manuals/SC3/man/SC3.pdf
CIDR^100^		R	248	https://github.com/VCCRI/CIDR
scDHA^35^		R		https://github.com/duct317/scDHA
ssCCES^37^		R		https://github.com/gedcom/scCCESS
DCA^36^		Python		https://scanpy.readthedocs.io/en/stable/generated/scanpy.external.pp.dca.html

**Table 3:** Non-AI pathway/geneset enrichment analysis tools.

Technique name	Version	Software platforms	# citations	Tutorial website
Seurat^30^ Ranksum	4.0	R, DAVID^34^	10,670	https://satijalab.org/seurat/reference/findallmarkersl
Seurat^30^ MAST	4.0	R, DAVID^34^	10,670	https://satijalab.org/seurat/reference/findallmarkers
Seurat^30^ NegBinom	4.0	R, DAVID^34^	10,670	https://satijalab.org/seurat/reference/findallmarkers
singleseqgset^101^		R	104	https://arc85.github.io/singleseqgset/articles/singleseqgset.html
ssGSEA^78^		R	13	https://ncborcherding.github.io/vignettes/escape_vignette.html
GSEA^20^	4.3.2		35,629	https://www.gsea-msigdb.org/gsea/doc/GSEAUserGuideFrame.html

**Table 4:** Non-AI scRNAseq trajectory analysis tools.

Technique name	Version	Software platforms	# citations	Tutorial website
Monocle^25^	3.0	R	4148	http://cole-trapnell-lab.github.io/monocle-release/docs/#constructing-single-cell-trajectories
PAGA^27^		Python	1579	https://scanpy-tutorials.readthedocs.io/en/latest/paga-paul15.html#Reconstructing-gene-changes-along-PAGA-paths-for-a-given-set-of-genes

Besides, we chose three recently-published tools: single-cell Decomposition using Hierarchical Autoencoder (scDHA)^35^, deep count autoencoder^36^ (DCA), and scCCESS^37^. In these tools, the Autoencoder was primarily for denoising the scRNAseq data, which may improve the cell clustering results. Therefore, these tools would be absent from the functional annotation analysis.

We also tried Geneset Enrichment Analysis (GSEA)^20^, a well-known pathway/geneset analysis but not built for scRNAseq data, via an ad-hoc experiment as follows. For each sample in the mouse dataset^14^, the average gene expressions over all cells were computed; then, these average expressions were treated as a ‘bulk-like’ expression. 8 samples were divided into ‘regenerative’ and ‘non-regenerative’ groups according to to^14^. In the pig dataset, the number of samples per group is small (between 1 and 3); therefore, synthetic sample data were created by randomly selecting 5000 cells from the same original sample. Then, the average gene expressions over all cells were calculated, being treated as a ’bulk-like’ sample. The group for synthetic samples was the same as the original sample. After preparing the ‘bulk-like’ data, the GSEA software and the MSigDB v2022.1^38^ was used to analyze the enriched pathways. GSEA software parameters were set by it default: number of permutations = 1000, metric to ranking genes = Signal2Noise, Maxsize: exclude larger set = 500, and Minsize: exclude smaller set = 15. The software will determine the enriched pathway; and if so, it will plot the pathway enrichment curve.

### Comparing methods evaluation

To evaluate the embedding and clustering performance between our proposed Autoencoder and other state-of-the-art techniques, the mouse heart scRNASeq data^14^ was used. These methods were applied to (i) visualize and isolate cardiomyocytes and (ii) recall two important mouse 'CM4' and CM5' cardiomyocyte subsets as reported in reference^14^ if (i) is successful. In^14^, cluster 'CM4' was explicit among regenerative and neonatal hearts: P1-MI-D1, P1-MI-D3, and P2-Sham. CM4 highly upregulated immature and cell-cycle markers Troponin I1 slow skeletal type (*Tnni1*), Ki-67 (*Mki67*) and Cyclin B1 (*Ccnb1*). CM5 was explicit among non-regenerative hearts: P8-MI-D1 and P8-MI-D3. CM5 highly upregulated hypertrophic marker Xin Actin-Binding Repeat Containing 2 (*Xirp2*) and cell adhesion marker *Cd44*. These markers were already validated using immunohistochemistry in reference^14^. Besides, the embedding and clustering results were evaluated in the much-larger human cardiac scRNAseq atlas^17^. Here, the tools were expected to replicate the identification of the major cardiac cell type clusters as in^17^:

- Ventricular cardiomyocytes were marked by clusters strongly expressing Titin (TTN), Cardiac Type Troponin T2 (TNNT2), Ryanodine Receptor 2 (RYR2), Myosin Heavy Chain 7 (MYH7), Myosin Light Chain 2 (MYL2), and Iroquois Homeobox 3 (IRX3).
- Atrial cardiomyocytes were marked by clusters strongly expressing TTN, TNNT2, RYR2, and Hepcidin Antimicrobial Peptide (HAMP).
- Cardiac endothelial cells were marked by clusters strongly expressing Cadherin 5 (CDH5), Platelet And Endothelial Cell Adhesion Molecule 1 (PECAM1), and Von Willebrand Factor (VWF).
- Cardiac pericytes were marked by clusters strongly expressing ATP Binding Cassette Subfamily C Member 9 (ABCC9) and Potassium Inwardly Rectifying Channel Subfamily J Member 8 (KCNJ8).
- Cardiac smooth muscle cells were marked by clusters strongly expressing Transgelin (TAGLN) and Smooth Muscle Actin Alpha 2 (ACTA2).
- The monocyte-macrophages were marked by clusters strongly expressing Macrophage-Associated Antigen CD163 and Lymphatic Vessel Endothelial Hyaluronan Receptor 1 (LYVE1).
- The lymphocytes were marked by clusters strongly expressing CD3 Epsilon Subunit Of T-Cell Receptor Complex (CD3E), CD3 Gamma Subunit Of T-Cell Receptor Complex (CD3G), and T-Cell Surface Glycoprotein CD8 Alpha Chain (CD8A).
- Cardiac fibroblasts were marked by clusters strongly expressing Decorin (DCN), Gelsolin (GSN), and Platelet Derived Growth Factor Receptor Alpha (PDGFRA).
- And the neuronal (glial) cells were marked by clusters strongly expressing Neurexin 1 (NRXN1), Neurexin 3 (NRXN3), and Potassium Calcium-Activated Channel Subfamily M Regulatory Beta Subunit 4 (KCNMB4).

The performances of the pathway/gene set enrichment analysis techniques were evaluated by assessing whether they recalled the upregulation of cell cycles among the P1-MI-D1 and P1-MI-D3 cells in reference^14^. Besides, we examined whether the techniques could demonstrate the upregulation of MAPK, HIPPO, cAMP, JAK-STAT, and RAS, which were upregulated in P1-MI mammals and validated in reference^39^. The statistical p-values of less than 10^–2^ were reported for statistical significance. Both the p-values and enrichment fold-changes were reported.

In the pig scRNAseq experiment, the technical performances were evaluated by reproducing the cardiomyocyte subpopulations that were validated in reference^40^. Briefly, a cardiomyocyte subpopulation, denoted 'CM1' (6537 cells), which was exclusive to the regenerative heart, was found. CM1 highly expressed proliferative regulators T-Box Transcription Factor 5 (TBX5) & T-Box Transcription Factor 20 (TBX20), Receptor tyrosine-protein kinase erbB-4 (ERBB4), and GRK5. Following the myocardial infarction on postnatal day P28, CM1 may primarily transit into two cardiomyocyte clusters, denoted 'CM2' and CM10'.

### Analyzing pigs’ cardiomyocytes with AI-based techniques when ^CCND2^hiPSC was injected into the injured heart

We combine ^CCND2^hiPSC-inject scRNAseq data with our previous embryonic, naïve, and MIp28-only scRNAseq data. This dataset was large and complex; therefore, multiple Autoencoder were built as follow:

- First, in the ^CCND^2hiPSC transplantation, an Autoencoder was built using the combined graft (hiPSC) and host (pig) cells. The original scRNAseq data were mapped to the combined human and pig reference genomes (GRCh38 and Sscrofa11.1) to quantify both human mRNA and pig mRNA. The Autoencoder and Uniform Manifold Approximation and Projection (UMAP)^41,42^ identified clusters where the count of human mRNA was significantly (tenfolds and more) than the count of pig mRNA; these clusters were human cells. Other clusters were pig cells.
- Second, after separating the pig cells from the human cells, the pig scRNAseq data were re-mapped and quantified only via the pig reference genome Sscrofa11.1. Another Autoencoder was built to cluster the pig cell types into cardiomyocytes, fibroblasts, endothelial cells, immune cells, and smooth muscle cells. Clusters explicitly expressing cardiomyocyte markers (ACTCT, MYH7, and RYR2) were separated (Supplemental Fig. 1).
- Third, the pig cardiomyocyte clusters in step 2 were combined, and this data was used to build another Autoencoder to cluster only cardiomyocytes (Supplemental Fig. 2).

After clustering the pig's cardiomyocytes, similar to^40^, the AI-based sparse support vector technique (sparse model) was applied to quantify cell-cycle phases and proliferative-supporting signaling pathways, including MAPK and HIPPO/YAP signaling. Here, Fetal cardiomyocytes were chosen as 'positive,' and CTL-P56 cardiomyocyte was chosen as 'negative' cells for computing the sparse model. We also applied other pathway & gene ontology enrichment analysis methods in Table 3 to identify which pathways were upregulated in the ^CCND2^hiPSC transplanted cardiomyocytes, compared to the control MIp28-only ones, on postnatal day 35 (7 days after the myocardial infarction injury).

We demonstrate the approach to separate the human cell from the pig cells by the following experiment. We used the raw SC sequencing data from two samples: one pig heart generated by our lab^18^, and the human-induced pluripotent stem cells (iPSC) from ArrayExpress, number E-MTAB-6687^19^. These data were mapped to the 'draft' pig Sscrofa10.2^20^ and the published human GRch38 genomes (http://www.ncbi.nlm.nih.gov/assembly/GCF_000001405.39/) using 10X Genomics CellRanger software version 3.1^24^. From CellRanger summary, it is clear that the pig transcripts can only be mapped to the pig genome (pig genome: 92.6%, human genome: 3.6%); meanwhile, the human transcripts can only be mapped to the human genome (pig genome: 28.2%, human genome: 95.6%). Also, we combined the human and pig housekeeping^25^ gene expression matrices from CellRanger and plot the cell clusters using UMAP^26^. Clearly, the human cells are completely separated from the pig cells in Supplemental Fig. 11. For pig cells, the ratio between map-to-pig-genome transcripts and map-to-human-genome is greater than 5 for all cells; meanwhile, this ratio is between 0 and 1 for all human cells.

Besides, since the proportion of cytokinesis cardiomyocytes is very low^29^, cells highly expressing cytokinesis-exclusive genes were counted to quantify and compare cytokinesis activity. The cytokinesis-exclusive gene list was chosen as follows. First, we obtained the gene participating in the cytokinetic process from Gene Ontology (GO) number GO:0032506^43^. Each gene may participate in more than one process described by GO terms. Therefore, for each gene in GO:0032506, we counted the number of cytokinesis-subprocess GO terms and non-cytokinesis terms, then calculated the ratio between these two numbers. Then, genes having this ratio of 0.9 or less were filtered out (Supplemental Table 1). Only AlkB Homolog 4, Lysine Demethylase (ALKBH4), Anillin, Actin Binding Protein (ANLN), Aurora Kinase B (AURKB), Centrobin- Centriole Duplication And Spindle Assembly Protein (CNTROB), and Kelch Domain Containing 8B (KLHDC8B) were considered cytokinetic-specific genes. Cardiomyocytes expressing at least 3 among these 5 genes in the scRNAseq data were considered cytokinetic cardiomyocytes.

## Results

**Figure 1:**
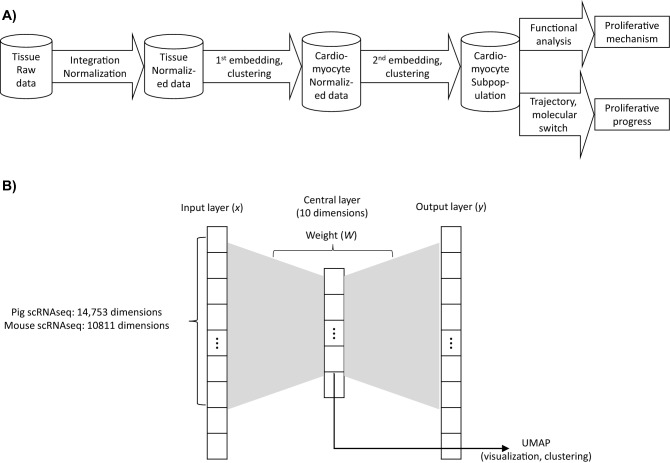
An AI-based approach for processing and interpreting scRNAseq datasets. (**A**) The scRNAseq analytical pipeline is displayed as a flowchart with the data processing steps in arrows and the input and output data for each step displayed as cylinders. (**B**) The architecture of the AI Autoencoder is displayed as a schematic.

After data integration and normalization, the scRNAseq analytic pipeline begins with two levels of embedding and clustering (Fig. 1A). The first level separates cardiomyocyte scRNAseq data from the data for other cell types, and the second level divides cardiomyocytes into subpopulations. Once the subpopulations are identified, pathway and gene set enrichment analysis is conducted to determine which cellular processes are up- or downregulated among the subpopulations, and trajectory analysis is conducted to determine whether one cell population may evolve into another over time and, if so, to identify genes that may trigger the transition between subpopulations.

### AI Autoencoder identified all major cardiac cell types in cluster analysis of scRNAseq data from mouse hearts, while non-AI techniques did not

Autoencoder^44^ is an AI technique that can synthesize and embed neural-network data^45,46^. It comprises at least three layers—an input layer, consisting of the original high-dimensional dataset, a central embedded layer with fewer dimensions, and a synthetic output layer whose dimensionality is equivalent to the input layer (Fig. 1B). Data from the input layer is alternately compacted into the embedded layer and then expanded to form the synthetic layer, and the computing sequence is optimized by minimizing the following function:

1 $$E=\frac{1}{N}\sum_{i}^{N}\sum_{j}^{N}{\left({x}_{i}-{y}_{j}\right)}^{2}+0.001{\Vert W\Vert }^{2}+Q$$

where *N* denotes the number of datapoints, *x*_*i*_ denotes an arbitrary input data point, *y*_*j*_ denotes an arbitrary synthetic datapoint, ||*W*||^2^ represents the regularization of Autoencoder weights, and *Q* represents the sparsity parameters^47^. After optimization, the output layer matches the input layer with maximum fidelity, and the embedded layer is considered an accurate low-dimensional representation of the input data.

Autoencoding can require a prohibitively large amount of computer memory^35,36,48^, which has prompted some researchers to reduce the dimensionality of the input data before the autoencoding procedure is initiated by (for example) including additional intermediate layers between the input and central layers and the central and output layers. However, the transcriptional heterogeneity of cardiac cells is high^49–51^ and likely further increased by the physiological changes that occur in response to cardiac injury. Thus, since reducing the dimensionality of the input data could mask this complexity, our AI Autoencoder retained the simple three-layered architecture, and the input layer was limited to genes with at least 1000 UMIs, yielding a dimensionality of 10,811 and 14,753 genes for the mouse and pig scRNA-seq datasets, respectively.

**Figure 2:**
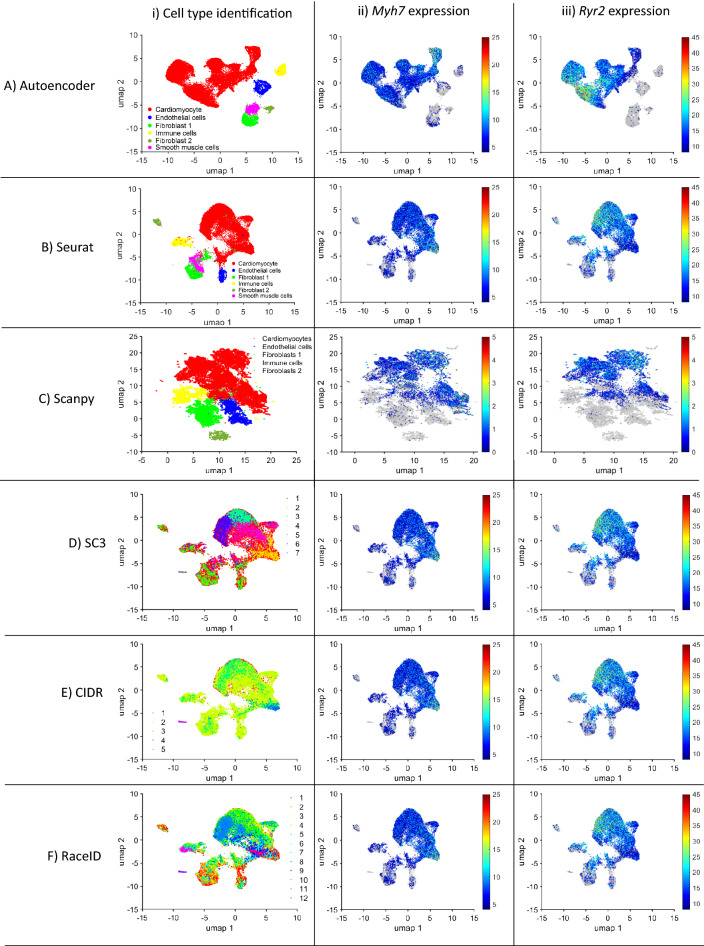

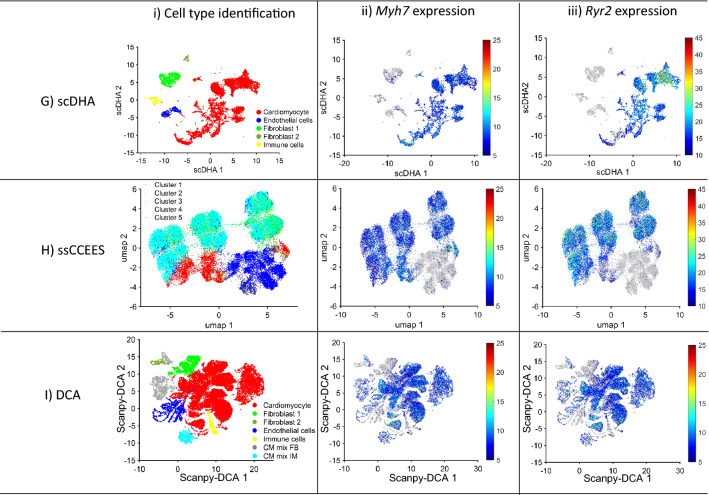
AI Autoencoder was more effective than non-AI tools for cluster analysis of scRNAseq data from mouse hearts*.* Cluster analysis of mouse heart scRNAseq data was conducted via (**A**) AI Autoencoder, (**B**) Seurat, (**C**) ScanPY, (**D**) SC3, (**E**) CIDR, (**F**) RaceID, (**G**) scDHA, (**H**) ssCCCEES or (**I**) DCA and (Column i) displayed via UMAP for identification of cell-type specific clusters. (Column ii) *Myh7* and (Column iii) *Ryr2* expression was quantified across the corresponding UMAP and presented as a heat map.

After AI Autoencoding, mouse data was visualized in two dimensions via Uniform Manifold Approximation (UMAP)^41,42^, the cells were clustered with the density-based clustering (dbscan)^52,53^ algorithm, and cell-type identity was determined via the expression of canonical markers for cardiomyocytes (*Myh7*, *Ryr2*^14^), fibroblasts (*Col1a1*, *Col1a2*^54^), endothelial cells (*Pecam1*, *Kdr*^55^), immune cells (*Bin2* and *Ifi30*^56^), and smooth muscle cells (SM22 alpha—*Tagln*^57^). When compared with five other highly cited clustering techniques (Seurat, ScanPY, SC3, CIDR, and RaceID), only Autoencoder and Seurat effectively generated cell-type–specific clusters (Fig. 2, Supplemental Fig. 1), whereas both *Myh7* and *Ryr2* were consistently and almost exclusively expressed by cells in the cardiomyocyte cluster of UMAPs from AI-Autoencoded data, a small but appreciable number of cells in the ScanPY cardiomyocyte cluster failed to express at least one of the two myocyte markers. Substantial myocyte marker expression was observed in all cell-type clusters generated via Seurat. Regarding ScanPY, cells expressing *Tagln* are scattered, making it difficult to identify smooth muscle cells. The other methods either failed to identify any cell types (SC3, CIDR, RaceID, ssCCEES—Fig. 2D–F,H), showed large clusters where the major cell types were mixed (DCA), or missing smooth muscle cell (ScanPY, DCA, scDHA—Fig. 2C,G,I). Importantly, each cell-type cluster included cells from all injury groups and time points (Supplemental Fig. 2), confirming that sample preparation variations did not compromise our results.

### AI Autoencoder distinguished cardiomyocyte subpopulations and their markers in injured and uninjured mouse hearts, while non-AI techniques missed important markers

The first published analysis^14^ of this mouse scRNAseq dataset identified five cardiomyocyte clusters (denoted CM1-CM5). One of the clusters (CM4) comprised > 20% of cardiomyocytes in animals that underwent MI or Sham surgery on P1, but just 4% in animals that underwent either procedure at P8, while a second cluster (CM5) was primarily observed in hearts that underwent MI rather than Sham surgery and was further enriched when MI induction was performed on P8. CM4 cardiomyocytes also appeared to re-enter the cell cycle after MI induction on P1 and expressed elevated levels of markers for cardiomyocyte immaturity (*Tnni1*), proliferation (*Mki67*), and cell-cycle activity (*Ccnb1*), while the hypertrophic marker *Xirp2* and the cell-adhesion molecule *Cd44* were upregulated in CM5 cardiomyocytes. Thus, cells in the CM4 cluster appeared to drive the regenerative response to MI induction on P1, while CM5 cardiomyocytes contributed to the adverse remodeling that occurred when MI was induced on P8.

**Figure 3:**
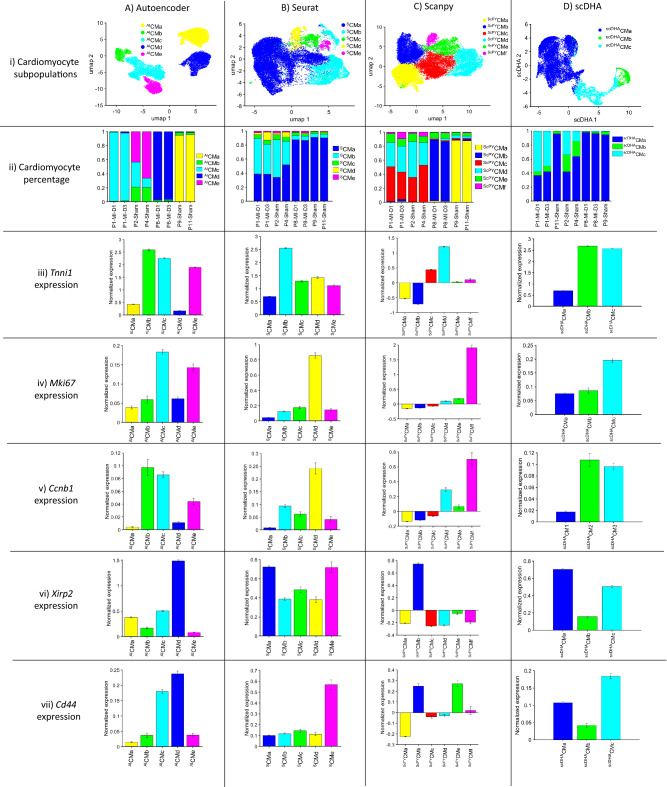
AI Autoencoder was more effective than non-AI tools for cluster analysis of cardiomyocyte scRNAseq data from mouse hearts. Cluster analysis of cardiomyocyte scRNAseq data was conducted via (**A**) AI Autoencoder (clusters ^AI^CMa-^AI^CMe), (**B**) Seurat (cluster ^S^CMa-^S^CMe), (**C**) ScanPY (clusters ^ScPY^CMa-^ScPY^CMf), or (**D**) scDHA (cluster ^scDHA^CMa-^scDHA^CMc) and displayed via (Row i) UMAP for identification of cardiomyocyte subpopulations. (Row ii) The proportion of cardiomyocytes from each cluster is displayed for each injury group and time point. (Rows iii-vii) The expression of (iii) *Tnni1*, (iv) *Mki67*, (v) *Ccnb1*, (vi) *Xirp2*, and (vii) *Cd44* was quantified for each cardiomyocyte cluster. Similarities between cluster labels are coincidental (e.g., clusters ^AI^CMa, ^S^CMa, and ^ScPY^CMa do not represent the same subpopulation). Expression data were normalized as in Seurat, briefly: the raw counts were logarithm (base 2) transformed and scaled according to the total of UMIs and detected genes per cell.

AI Autoencoder largely replicated these results, but with even greater specificity for injury group and time point. Of the five AI-Autoencoder–identified cardiomyocyte subpopulations (^AI^CMa-^AI^CMe, Fig. 3Ai), ^AI^CMc comprised > 95% of cardiomyocytes in hearts from P1-MI-D1 and -D3 animals and 10–35% of cardiomyocytes in P1-Sham-D1/D3 animals but were largely absent in animals that underwent surgical procedure on P8. In contrast, the ^AI^CMd cluster included > 95% of cardiomyocytes in P8-MI-D1/D3 animals and no more than 5% from any other group or time point (Fig. 3Aii). *Tnni1*, *Mki67*, and *Ccnb1* also tended to be upregulated in the ^AI^CMc cluster as well as in ^AI^CMb and ^AI^CMe cardiomyocytes (Fig. 3Aiii-v), which together comprised the majority of cardiomyocytes in P1-Sham-D1/D3 hearts, while both *Xirp2* and *Cd44* were highly expressed in ^AI^CMd (Fig. 3Avi,vii). Seurat clustering also identified five cardiomyocyte subpopulations (^S^CMa-^S^CMe, Fig. 3Bi); however, the distribution of cardiomyocytes across the five clusters differed somewhat between P1- and P8-operated animals. It did not vary substantially between injury groups (Fig. 3Bii). For example, the ^S^CMb cluster was enriched in both P1-MI-D1/D3 and P1-Sham-D1/D3 animals, where it included 30–50% of all cardiomyocytes compared to less than 10% in P8-MI- and P8-Sham-D1/D3 animals, while the ^S^CMa cluster comprised a much larger proportion of cardiomyocytes in both P8-MI- and P8-Sham-D1/D3 hearts (> 85%) than in P1-operated hearts (30–55%). Furthermore, although ^S^CMb cardiomyocytes were more common in the hearts of younger animals, they did not appear to express elevated levels of *Mki67* or *Ccnb1* (Fig. 3Biv-v). ScanPY, where DCA was co-executed, identified six cardiomyocyte clusters (^ScPY^CMa-^ScPY^CMf, Fig. 3Ci), two of which were found almost exclusively in P8-MI-D1/D3 (^ScPY^CMb) or P8-Sham-D1/D3 (^ScPY^CMa) hearts, where they comprised more than 80% of all cardiomyocytes. ScanPY also identified two clusters (^ScPY^CMc and ^ScPY^CMd) that together comprised 75%-85% of cardiomyocytes in P1-MI or P1-Sham animals and were largely absent in animals that underwent either surgery on P8; however, the distribution of cardiomyocytes across clusters in P1-operated animals was largely similar, regardless of injury group (Fig. 3Cii), and neither ^ScPY^CMc nor ^ScPY^CMd cardiomyocytes displayed evidence of *Mki67* upregulation (Fig. 3Civ). Collectively, these observations demonstrate the clear difference between the AI Autoencoder and non-AI clustering techniques for identifying cardiomyocyte subpopulations associated with the regenerative response to MI induction in mouse hearts; furthermore, the AI Autoencoder clustering results demonstrated the upregulation of all cell-cycle markers, which were missed by non-AI techniques.

On the other hand, scDHA identified three cardiomyocyte clusters (^scDHA^CMa-c). Cluster scDHACMc were more enriched among the regenerative groups PI-MI-D1/D3 (Fig. 3D-ii), co-upregulated cell cycle markers *Tnni1*, *Mk67*, *Ccnb1* (Fig. 3D-iii-v), but also co-upregulated hypertrophy marker *Xirp2* and *Cd44* (Fig. 3D-vi-vii). Therefore, although scDHA, which also utilized Autoencoder, could identify a cardiomyocyte cluster strongly presented in the regenerative-heart groups, the method failed to differentiate whether this cluster demonstrated a proliferative response or a hypertrophic response.

### AI sparse modeling identified cardiomyocytes with upregulated cell-cycle and pathway activity, whereas non-AI techniques did not

Conventional techniques for pathway and gene set enrichment analysis^58^ begin with a list of differentially expressed genes between cell populations and then infer which cellular processes or pathways are up- or down-regulated between populations. In AI Sparse Modeling^59–62^, these two steps are implemented in the reverse order, beginning with sets of genes that are known to participate in the process being studied and then determining whether these genes and their associated pathways are differentially activated in the cells. Thus, whereas the conventional approach can only be applied to cell populations (or subpopulations), AI Sparse Modeling can be used to evaluate the data for an individual cell and extract relevant information from datasets containing a large number of variables that do not contribute to the property being studied. However, the technique requires designating predefined "positive" and "negative" cell groups, so since the proliferative activity of cardiomyocytes in neonatal mice declines precipitously during the first several days after birth, cardiomyocytes from P1-Sham-D1 (collected from 2-day-old mice) and P8-Sham-D3 animals (collected from 11-day-old mice) were designated positive (i.e., proliferating) and negative (i.e., nonproliferating), respectively.

The sparse model estimates a score *y* for each cell expression data vector **x** via the linear formula:

2 $$y=\mathbf{w}\mathbf{x}+b$$

**x** denotes the gene expression vector, **w** denotes the coefficient for each gene in a pathway or geneset, and the parameters **w** and *b* are computed by minimizing

3 $$\frac{1}{2}\left|\mathbf{w}\right|+C\sum_{\forall i}{\epsilon }_{i}$$

subject to

4 $$\left\{\begin{array}{l}{y}_{i}\left(\mathbf{w}{\mathbf{x}}_{i}+b\right)+{\epsilon }_{i}\ge 1\\ {\epsilon }_{i}\ge 0\end{array} \forall i\right.$$

where ${\epsilon }_{i}$ represents the accuracy of Eq. (2) when applied to cell *i*, with a smaller ${\epsilon }_{i}$ indicating greater accuracy. **w** and *b* were initially calculated for cardiomyocytes in the positive and negative groups, with *y* = 1 and *y* = − 1, respectively, and then used to calculate y (Eq. 2) for all other cardiomyocytes. Cells with *y* > 1 were categorized "high," cells with *y* < –1 were categorized "low," and cells with − 1 $\le$ *y* $\le$ 1 were categorized "middle." Thus, a "high" categorization (for example) indicated that the cell was more similar to P1-Sham-D1 than to P8-Sham-D3 cardiomyocytes and, consequently, more likely to be proliferative.

**Figure 4:**
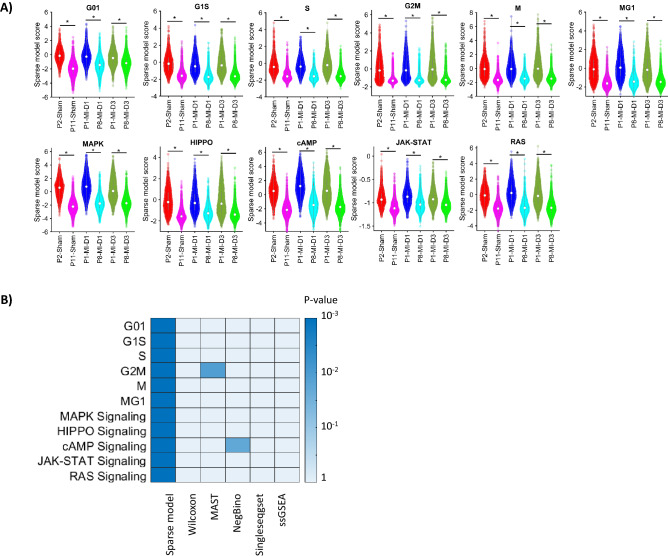
AI Sparse Modeling was more effective than non-AI tools for pathway/gene set enrichment analysis of cardiomyocyte scRNAseq data from mouse hearts. (**A**) Sparse model scores for each cell-cycle phase and for the activity of the MAPK, HIPPO, cAMP, JAK-STAT, and RAS signaling pathways were summarized for cardiomyocytes from the indicated injury groups and time points and presented as violin plots. Statistical comparison between the P1-MI-D1/D3 (regenerative) and P8-MI-D1/D3 (non-regenerative), also between P2-Sham and P11-Sham, were done by non-parametric tests; *p-value < 0.01. (**B**) Cardiomyocytes from the P1-MI-D3 and P8-MI-D3 groups were scored for cell-cycle phase and pathway activity via AI Sparse Modeling, Wilcoxon Ranksum test, MAST, Negative Binomial (NegBino) test, Singleseqset, and ssGSEAP; then, the scores generated by each technique were compared between time points, and the P-values for each comparison was presented as a heat-map.

Analyses were conducted for the cell-cycle markers^14^ and genes associated with MAPK^63^, HIPPO^64^, cAMP^65^, JAK-STAT^66^, and RAS^67^ signaling (Fig. 4A), which are known to be upregulated in the mammalian hearts that underwent MI on P1^39^. The AI Sparse Model identified statistically significant differences (P < 0.01) between cardiomyocytes from the Regenerative P1-MI-D1/D3 and Non-regenerative P8-MI-D1/D3 groups for all cell-cycle phases and all signaling pathways. At the same time, only a single parameter differed significantly when the data were analyzed via Seurat MAST (G2-M phase transition) or Seurat Negbinom (cAMP signaling), and three other highly cited non-AI techniques (Wilcoxon Ranksum, Singleseqgset, and ssGSEA) failed to identify any significant differences between groups (Fig. 4B). Beside, GSEA (the data availability section) did not identify cell cycle and other signaling pathways. Overall, AI Sparse Modeling identified most of the previously-validated genes and pathways may be differentially activated in proliferating and non-proliferating cardiomyocytes; meanwhile, the non-AI techniques missed many of these genes and pathways.

### AI semisupervised learning identified transformation among cardiomyocyte subpopulations, where non-AI trajectory analysis did not

The pig scRNA-seq dataset analyzed for this report was generated in our double-injury model. Animals underwent AR_P1_, MI_P28_, both AR_P1_ and MI_P28_ (AR_P1_MI_P28_) or neither myocardial injury (CTL), and assessments conducted on P56 indicated that the hearts of animals in the MI_P28_ group displayed significant fibrosis and declines in contractile activity, AR_P1_MI_P28_ animals completely recovered with no evidence of myocardial scarring or loss of cardiac function. The complete cardiac scRNAseq dataset included data from AR_P1_ animals on P28 and P56; from MI_P28_ and AR_P1_MI_P28_ animals on P30, P35, P42, and P56; from AR_P1_MI_P28_ animals on P30, P35, P42, and P56; from CTL animals on P1, P28, and P56; and fetal pigs. AI Autoencoding identified 10 cardiomyocyte clusters (denoted CM1-CM10), one of which (CM1) comprised 62.91% of the cardiomyocytes present in AR_P1_ hearts on P28 but was essentially absent in all other injury groups and at all other timepoints. In comparison, two other clusters (CM2 and CM10) collectively encompassed 89.62% of cardiomyocytes in AR_P1_MI_P28_ hearts on P30^40^. Notably, CM1 cardiomyocytes were also enriched for the expression of three genes (TBX5^68,69^, TBX20^70,71^, and ERBB4^72^) that contribute to the proliferation of cardiomyocytes in fetal and neonatal mouse hearts. Collectively, these observations suggest that AR on P1 preserved some neonatal-like proliferative capacity in a subpopulation of cardiomyocytes that subsequently formed the CM1 cluster, and that MI on P28 triggered this latent proliferative capacity, thereby driving the transformation of CM1 cardiomyocytes into either CM2 or CM10 cardiomyocytes.

Trajectory analysis via our AI Semisupervised learning model^73,74^ tracks the transformation of one type of cell (e.g., CM1) into two other cell types (e.g., CM2 and CM10), via a procedure that is analogous to AI Sparse Modeling for pathway and geneset enrichment, with the two endpoints of the transformational trajectories serving as the predefined positive (CM2, *y* = 1) and negative (CM10, *y* = − 1) cells. The **w** coefficients and *b* parameters were initially computed by applying formulas 2–4 to the cells at the endpoints of the trajectories (CM2 and CM10) and then used to calculate *y* (via formula 2) for each cell at the beginning of the trajectory (CM1). CM1 cells for which *y* > 0.1 were categorized as CM1→2, CM1 cells for which *y* < − 0.1 were categorized as CM1→10, and all other CM1 cells were categorized as inconclusive; then, CM2 cells were combined with CM1→2 cells (CM2 + 1→2), CM10 cells were combined with C1→10 cells (CM10 + 1→10), **w** and *b* were re-computed via formulas 2–4 with the combined cell populations serving as the predefined positive (CM2 + 1→2, *y* = 1) and negative (CM10 + 1→10, *y* = − 1) cell populations, y was recalculated for CM1 cells, and the procedure was repeated until the CM1→2, CM1→10, and inconclusive categories did not change. The results from our AI Semisupervised Learning Model^40^ indicated that most (84.78%) CM1 cardiomyocytes would likely follow the CM1→2 trajectory, while the remainder (15.22%) followed the CM1→10 trajectory^40^.

**Figure 5:**
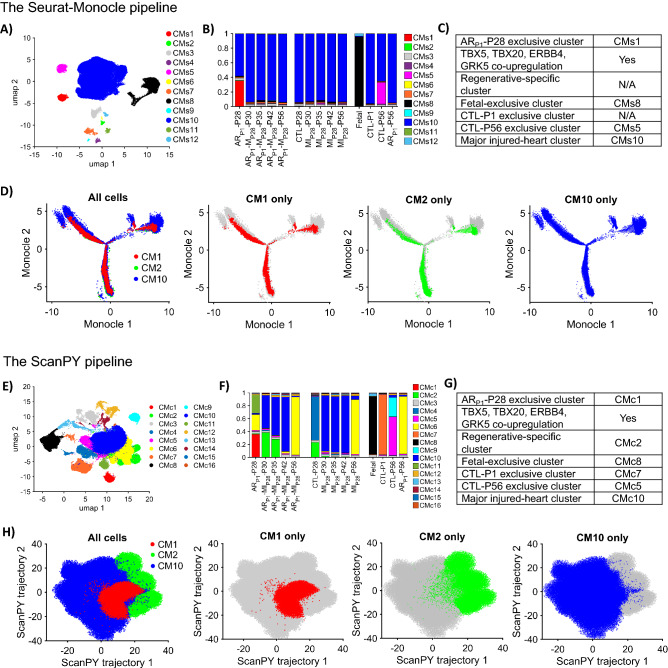
AI semisupervised learning was more effective than non-AI tools for trajectory analysis of cardiomyocyte scRNAseq data from pig hearts (**A**) Seurat-Monocle pipeline UMAP plot visualizing 12 cardiomyocyte clusters (CMs1-CMs12), where these clusters were identified by Seurat pipeline^30^. (**B**) The proportion of cardiomyocytes from each cluster CMs1-CMs12 is displayed for each injury group and time point. (**C**) Summary of clusters resulting from the Seurat-Monocle pipeline that are: exclusive for ARp1-P28, Fetal, CTL-P1, CTL-P56, and co-upregulation of TBX5/TBX20/ERBB4/GRK5, which do not show any clusters explicit for ARP1-MIP28 cardiomyocytes on P30, P35, and P42. (**D**) Seurat-Monocle pipeline trajectory plot among CM1, CM2, and CM10, whereas CM1, CM2, and CM10 were defined in^40^. (**E**) ScanPY pipeline UMAP plot visualizing 16 cardiomyocyte clusters (CMc1-CMc16) identified by ScanPY. (**F**) The proportion of cardiomyocytes from each cluster CMc1-CMc12 is displayed for each injury group and time point. (**G**) ScanPY pipeline, a summary of clusters that are: exclusive for ARp1-P28, Fetal, CTL-P1, CTL-P56, and co-upregulation of TBX5/TBX20/ERBB4/GRK5. (**H**) ScanPY pipeline trajectory plot among CM1, CM2, and CM10. For comparison, the figures for the AI-based method were available at^40^.

The combined Seurat–Monocle pipeline (Fig. 5A–D) found 12 cardiomyocyte clusters (CMs1-CMs12). Among them, an ARp1-P28-exclusive CMs1 cardiomyocyte cluster co-upregulated TBX5, TBX20, ERBB4, and GRK5, Fetal-exclusive CMs8, CTL-P56-exclusive CMs5. However, this pipeline could not identify any cluster exclusive for CTL-P1 cardiomyocytes (Fig. 5C). Therefore, we replaced Seurat cluster result^40^ by the AI cluster prior to Monocle. Although Monocle revealed a three-branch trajectory, none of the three branches were explicit for either CM1, CM2, or CM10 (Fig. 5D). Therefore, the combined Seurat-Monocle pipeline was unlikely to tell how the highly regenerative-potential ARp1-P28 cardiomyocytes evolved following MIp28 injury.

Meanwhile, ScanPY pipeline (Fig. 5E–H) resulted in 16 cardiomyocyte clusters (CMc1-CMc16). ScanPY UMAP visualization showed overlapping and 'breaking up' cardiomyocyte clusters, such as CMc7 and CMc2 were visualized by multiple blocks. Still, ScanPY also found cluster CMc1 exclusive for ARp1-P28 and co-upregulated TBX5, TBX20, ERBB4, and GRK5. Cluster CMc2 only appeared in regenerative ARp1-P28, ARp1-P28-P30, and ARp1-P28 groups and upregulated the same markers to the AI-found cluster CM2. Also, exclusive clusters for Fetal (CMc8), CTL-P1 (CMc7), and CTL-P56 (CMc5 and CMc9) were founded. Cluster CMc10 covered the majority of injured-heart cardiomyocytes. However, the ScanPY trajectory result (Fig. 5H) did not show any clear trajectory among CMc1, CMc2, and CMc10.

### AI-based Autoencoder, Seurat, and ScanPY identified major cell types in the large human cell atlas dataset

**Figure 6:**
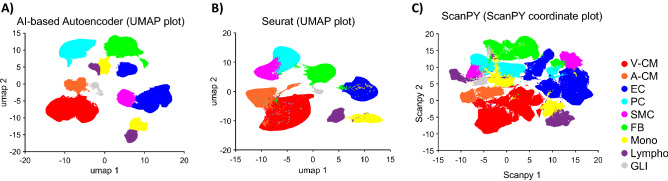
Our proposed pipeline, Seurat, and ScanPY can analyze and identify all major cell types in the human heart cell atlas data. 2D plots visualize the cell clustering and cell-type in (**A**) AI-based Autoencoder (UMAP plot), (**B**) Seurat (UMAP plot), (**C**) ScanPY (ScanPY coordinate plot). V-CM (red): ventricular cardiomyocyte. A-CM (ligh red): atrial cardiomyocyte. EC (blue): endothelial cell. PC (cyan): pericyte. SMC (pink): smooth muscle cell. FB (green): fibroblast. Mono (yellow): monocyte and macrophage. Lympho (violet): lymphocyte, including T cells and natural killer cells. GLI (grey): neuronal (glial) cell.

Figure 6 visualizes the cell-clustering results among our AI-based Autoencoder (Fig. 6A), Seurat (Fig. 6B), and ScanPY (Fig. 6C) when analyzing 486,134 human cardiac cells. In these methods, the cardiomyocytes form a large, isolated cluster; furthermore, the separation between ventricular cardiomyocyte and atrial cardiomyocyte can be clearly seen. The cluster-to-cell-type assignment is consistent with the cell-type-specific marker-expression localization (Supplemental Figs. 5, 6, 7, 8). Other methods listed in Table 2 failed to execute the large-scale (486,134 cells) human data and were absent from Fig. 6.

### AI-based Autoencoder outperforms PCA dimensional reduction in representing and restructuring the data

Due to a large number of genes (high dimension), most scRNAseq analytic pipelines perform dimensional reduction before clustering and embedding data. In Seurat^30^ and ScanPY^75^, Principal Component Analysis (PCA) is used for dimensional reduction. In principle, the high-dimensional original scRNAseq data was transformed into lower-dimensional data. The lower dimensional data can reconstruct the original data; furthermore, good dimensional reduction should reconstruct the data similarly to the original ones. Therefore, we compare the reconstructed-original data similarity:

5 $$S=\frac{1}{N}\sum_{i}^{N}{\left({x}_{i}-{y}_{i}\right)}^{2}$$

In formula (5), *x*_*i*_ denotes an arbitrary original cell, and *y*_*i*_ denotes the reconstructed cell, which is computed from *x*_*i*_ by the Autoencoder or PCA. Lower *S* implies more similarity. In Table 5, we compared reconstructed-original data similarity between the Autoencoder, PCA (using all reduced features) and PCA2000 (using only the best 2000 reduced features as in Seurat) for each pig heart^40^ (GEO database accession number GSE185289). Clearly, Autoencoder achieves a higher degree of similarity in all hearts except 8060_AZ, which means that the Autoencoder 10 embedded features represent the scRNAseq data more accurately than 2000 PCA features.

**Table 5:** Comparing reconstructed-original data similarity (*S* score) among Autoencoder, PCA2000 and PCA approach.

SampleID	Autoencoder	PCA 2000	PCA (all features)
7995_BZ	**40.18**	43.27	41.22
8014_BZ	**40.16**	44.13	42.44
8015_BZ	**40.73**	43.90	41.92
8026_BZ	**40.71**	42.97	41.04
8026_P1	**41.29**	47.40	45.25
8030_CZ	**40.79**	44.60	42.95
8046_BZ	**40.21**	44.99	43.72
8052_AZ	**38.80**	43.30	41.38
8060_AZ	42.98	40.04	**37.72**
8060_IZ	**41.08**	42.24	41.23
8064_AZ	**39.27**	45.88	44.18
8064_CZ	**39.26**	42.87	41.21
8094_AZ	**40.70**	44.36	41.96
8095_AZ	**39.87**	43.36	41.51
8095_BZ	**39.99**	42.52	40.84

The lowest *S* score (most reconstructed-original similarity) approach is bold-highlighted.

### Different clustering algorithms can produce consistent cell type identification if using AI-based Autoencoder embedded layer

Since our cell clustering approach involves on the Autoencoder embedding, clustering algorithms, and visualization, it is interesting to examine whether the embedding primarily determine the clustering results. Therefore, we applied different clustering algorithms: the 'simple' K-mean^76^, the Louvain^77^, and density-based (dbscan) algorithms^52^ to cluster our previous pig scRNAseq data^9,40^ after the dataset was encoded (embedded) into just 10 dimensions. Visualizing the clustering results (Supplemental Fig. 9) using the UMAP and cell type markers from our previous work^40^, it is interesting seeing that the K-mean clustering (K = 7, Supplemental Fig. 9A), Louvain (implemented according to https://github.com/GenLouvain/GenLouvain, Supplemental Fig. 9B), and dbscan (implemented according to https://www.mathworks.com/help/stats/dbscan.html with epsilon = 0.2 and minpts = 50, Supplemental Fig. 9C) show very consistent results. Furthermore, these results were nearly identical to our previous report in^40^, where dbscan were performed on the UMAP visualization instead of the embedding. Since very different clustering algorithms produced very similar results, the cell type identification in our pipeline is mostly determined by the embedding.

### AI-based techniques found increased pigs' cardiomyocyte proliferation and upregulated HIPPO/YAP & MAPK signaling pathways 7 days after ^CCND2^hiPSC injection

**Figure 7:**
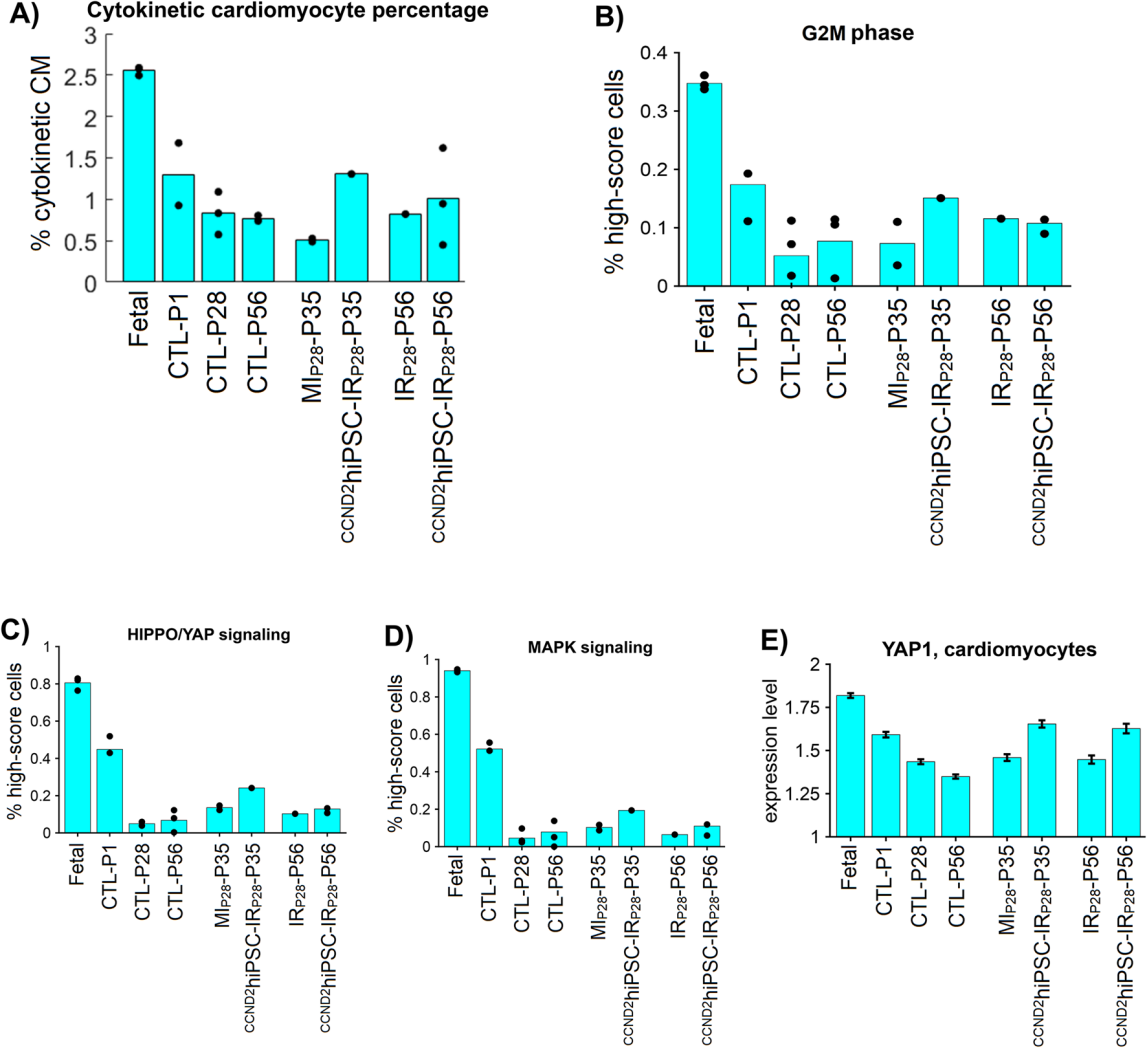
Single-nuclei RNA sequencing shows that ^CCND2^hiPSC-MI cardiomyocytes increase cycling and upregulated HIPPO/YAP & MAPK signaling pathways, especially 7 days after MI injury. (**A**) Percentage of cardiomyocytes highly expressing cytokinesis-specific genes AURKB, ALKBH4, ANLN, CNTROB, and KLHDC8B in each group. (**B**–**D**) Bar graphs: sparse analysis quantifies the G2M phase, HIPPO/YAP, and MAPK signaling pathways in each heart; here, the sparse model only used DNA synthesis genes to compute a 'sparse model score' for each cell such that the score optimally separates fetal from naïve-P56 cardiomyocytes; a higher score implies more active G2M, HIPPO/YAP and MAPK activities. Each dot is the percentage of cells having 'high model score' in a heart. (**E**) Error bar: YAP1 average expression, which was per cell in each group; here, the raw counts were logarithm (base 2) transformed and scaled according to the total of UMIs and detected genes per cell by Seurat^30^.

In Fig. 7, the percentage of cytokinetic cardiomyocytes is the highest in the fetal heart (2.55%), then it gradually decreases through CTL-P1 (1.30%), CTL-P28 (0.83%), and CTL-P56 (0.76%) cardiomyocytes. This decrease is consistent with the fact that wildtype cardiomyocyte proliferation gradually shutdowns 7 days after birth in mammals^4^; it also validates our cytokinetic-specific markers and quantification method. Interestingly, the percentage of cytokinetic cardiomyocytes increases in ^CCND2^hiPSC-IR_P28_-P35 (7 days after injection) to 1.30%, which is close to the CTL-P1 level, then decreases to 0.96% on ^CCND2^hiPSC-IR_P28_-P56. Meanwhile, this percentage does not increase in the control MI_P28_-P35 heart (0.51%). Furthermore, the sparse model analysis showed that ^CCND2^hiPSC-P35 increased G2 to Mitosis and Mitosis cell cycle activities. Concurrently, HIPPO/YAP & MAPK signaling pathways, which are known to be associated with cardiomyocyte proliferation, were upregulated in ^CCND2^hiPSC-P35 compared to MI_P28_-P35 cardiomyocytes. Furthermore, YAP1 expression in ^CCND2^hiPSC-IR_P28_-P35 was elevated. Together, these results suggest that ^CCND2^iPSC injection may communicate with and promote the hosts' cardiomyocyte proliferation through the HIPPO/YAP and MAPK pathways.

Meanwhile, Seurat-NegBino (Supplemental Fig. 10A) and Seruat-MAST (Supplemental Fig. 10B), which do not calculate the pathway enrichment for individual cells, analyzed differentially-expressed genes in ^CCND2^hiPSC-IR_P28_-P35 cardiomyocytes; they showed that these genes enrich TGF-beta and JAK-STAT signaling pathways. None of the cell-cycle biological processes were found enriched in these methods. On the other hand, our AI-based sparse model and ssGSEA^78^ can compute the enrichment score in each cell. The sparse model analysis shows that ^CCND2^hiPSC-IR_P28_-P35 cardiomyocytes have higher scores for cell-cycle G1 to DNA synthesis (G1S)^79^, DNA synthesis (S)^80^, G2 to Mitosis (G2M)^81^, and cytokinesis stages (Supplemental Fig. 10C–F). Meanwhile, ssGSEA only shows that ^CCND2^hiPSC-IR_P28_-P35 cardiomyocytes have higher enrichment score for G1S and cytokinesis stages (Supplemental Fig. 10C–F). In addition, the sparse model shows that ^CCND2^hiPSC-IR_P28_-P35 cardiomyocytes increase MAPK, HIPPO, and TGFβ signaling pathways; meanwhile, ssGSEA found that the ^CCND2^hiPSC-IR_P28_-P35 cardiomyocyte enriched cAMP, RAS, and TGFβ signaling pathways (Supplemental Fig. 10G–L). Overall, the sparse model results showed the highest number of upregulated cell-cycle stages in ^CCND2^hiPSC-IR_P28_-P35, and it was the only method identifying the HIPPO signaling pathway.

## Discussion

The field of cardiovascular science has been actively generating scRNAseq datasets^82^. The large-scale cardiac scRNAseq data (> 10,000 cells) first appeared in 2018^18^, then massive-scale (> 200,000 cells) datasets^17^ emerged, including our previous works in^9,40^. In the field, the most important objective is to find cell-type subpopulations that are specific for the disease or phenotype and occur at specific time-windows. These subpopulations are rare^82^; therefore, analyzing cardiac scRNAseq emphasizes the precision in detecting the rare subpopulations over the computing burden. In this report, two large-scale and two massive-scale scRNAseq datasets from mouse, pig, and human were analyzed and compared among the analytic pipelines. Our proposed pipeline rediscovered all phenotype (proliferation and hypertrophy) and timepoint-specific cardiomyocyte subpopulations that were validated in the previous works^9,14,40^. Furthermore, the pipeline identified cardiomyocyte-proliferation regulators upregulated in the host (pig) cardiomyocytes when the engineered hiPSC-derived cardiomyocytes (graft) were transplanted into an injured heart model. These findings serve the most important objective of using scRNAseq data in cardiovascular science. The pipeline can be applied to analyze non-cardiac scRNAseq data, when detecting ‘rare’ cell subpopulations and stages is the priority.

The fundamental goal of regenerative myocardial therapy is to replace the scarred region of infarcted hearts with functional contractile tissue. Many of the strategies that are currently under investigation involve the delivery of cells or engineered tissues to the infarcted region^29,83–85^; however, despite substantial advancements in both approaches, engraftment rates remain unacceptably low, and whether the transplanted cells adequately couple with the native myocardium has yet to be conclusively determined. Techniques for inducing the proliferation of endogenous cardiomyocytes alleviate both concerns because the engraftment process is no longer relevant, and coupling is likely to be more extensive between daughter cardiomyocytes generated via the division of a parent cell than between endogenous and transplanted cardiomyocytes. Thus, by generating a comprehensive list of molecules and signaling mechanisms that regulate myocardial regeneration, the AI scRNAseq toolkit presented here could develop a transformative approach to treating cardiac disease.

All three components of scRNAseq analysis (clustering, pathway/geneset enrichment, and trajectory analysis) were executed more effectively when the data were processed via our AI scRNAseq toolkit than via non-AI techniques. AI Autoencoder was the only tool to identify substantial differences between the cardiomyocyte subpopulations that comprised P1-MI and P1-Sham mouse hearts on D3, especially the ones corresponding to proliferative and hypertrophic responses; statistically significant differences between cardiomyocytes from the P1-MI-D1/D3 and P8-MI-D1/D3 mouse groups were identified for six cell-cycle phases and five signaling pathways when the data were analyzed via AI Sparse Modeling, compared to a total of just one cell-cycle phase and one pathway when the data were analyzed via five non-AI techniques; and whereas the non-AI techniques failed to detect any potential transformational changes among the CM1, CM2, and CM10 clusters in pig hearts, AI Semisupervised Learning found two distinct subpopulations of the CM1 cluster that were primed to follow the CM1→2 and CM1→10 trajectories. Notably, ten other highly cited scRNAseq tools (BackSPIN^86^, SPADE^87^, RCA^88^, SIMLR^89^, URD^90^, SCope^91^, SNN-Cliq^92^, TSCAN^93^, SCDE^94^, and Slingshot^26^) failed to complete the analyses without generating technical errors, perhaps because they were likely developed and tested on datasets that were much smaller than those used in this report. Furthermore, our pipeline was the only one identifying the upregulation of the HIPPO signaling pathway, a critical cardiomyocyte proliferation regulator^95,96^, among the host cardiomyocytes following a transplantation treatment, which directly explain the host cardiomyocyte proliferation observed in^29^. Thus, our AI-based approach is more effective than many non-AI scRNAseq tools for analyzing the immense datasets needed to accommodate the vast heterogeneity of cardiac cells—particularly cardiomyocytes—in the hearts of animals that are recovering from myocardial injury. However, to analyze the massive-scale dataset, our AI-based toolkit was implemented with proprietary software (Matlab) in the current study and required more than 50 GB of computer memory and approximately 24 h of processing time for a 10,000-gene dataset, which limits its compatibility with standard lab computers. Methods to reduce the computing burden, including training the Autoencoder using a smaller (~ 3000 genes) but representative gene list and using transfer learning^97^, will be examined in future works.

This work also reported the performance of other utilizing-Autoencoder pipelines^35–37^ in analyzing cardiac scRNAseq data. In these pipelines, the primary task for Autoencoder is data denoising; meanwhile, in our pipeline, Autoencoder's primary task is data embedding. Among them, ssCCES could identify cardiomyocyte subclusters, but failed to separate other cardiac cell types. Meanwhile, DCA, which was integrated into ScanPY, did not help ScanPY improve the cluster cell type identification step in the mouse dataset; rather, applying DCA resulted in clusters mixing multiple cell types (cardiomyocyte-mix-fibroblast and cardiomyocyte-mix-immune cells). On the other hand, while scDHA identified a cardiomyocyte cluster explicit for the regenerative-heart group (^scDHA^CMc), this cluster upregulated both proliferative and hypertrophy markers; therefore, it was unable to separate proliferation from hypertrophy, which is a fundamental requirement in cardiac regeneration. These pipelines' results suggest that although Autoencoder data denoising was effective in other non-cardiac scRNAseq data, its performance was very limited in cardiac scRNAseq data. One explanation for this failure is that cardiomyocyte proliferation, marked by expression of AURKB and a few other genes, is a rare event. In our manuscript, counting from the single-cell data, the percentage of AURKB + cardiomyocytes is only at most 2–3%, which also means no more than 1.5% of the overall cardiac scRNAseq data. The small percentage of AURKB + cardiomyocyte was also reported by other works^1,3^. Due to the very small percentage, denosing methods may mistakenly consider these critical proliferating cardiomyocytes as 'noise'; therefore, they may miss important results about cardiomyocyte proliferation.

In conclusion, for the cluster, pathway/gene set enrichment, and trajectory analysis of scRNAseq datasets generated from studies of myocardial regeneration in mice and pigs, our AI-based toolkit identified results that non-AI techniques did not discover. These different results were validated and were important in explaining myocardial regeneration. Ongoing work will adapt the toolkit for implementation with open-source software (e.g., R or Python) and improve the toolkit's compatibility with standard laboratory computers by investigating methods for reducing dimensionality, such as the inclusion of intermediate layers in the AI Autoencoder architecture.

## Supplementary Information

Supplementary Information.

## Data Availability

The source codes for AI techniques are publicly available at https://github.com/thamnguy/Cardiac-single-cell-AI, with a detailed tutorial at https://sites.uab.edu/jayzhanglab/products/ai-pipeline/. A R-version replicate can be found at https://sites.uab.edu/jayzhanglab/ai-pipeline-r/. This work used the publicly available scRNAseq data at Gene Expression Omnibus accession numbers GSE130699 and GSE185289. The GSEA analysis result is available at https://github.com/thamnguy/Cardiac-single-cell-AI/tree/main/GSEA%20analysis. The new ^CCND2^hiPSC-inject scRNAseq data will be publicly available when the manuscript is accepted for publication.
