# Supplementary material for: Analysis of cardiac single-cell RNA-sequencing data can be improved by the use of artificial-intelligence-based tools
Source: Sci Rep. 2023 Apr 26;13:6821. doi: 10.1038/s41598-023-32293-1 (PMC10133286; doi:10.1038/s41598-023-32293-1)
Supplement: Supplementary file 1 — Supplementary Information. [file 41598_2023_32293_MOESM1_ESM.pdf]

Supplemental Figure 1

UMAP plot of all heart cells after Autoencoder clustering, cell localization of the groups (Fetal, CTL-P1, CTL-P28, CTL-P56, MI_{P28}-P35, ^{CCND2}hiPSC-IR_{P28}-P35, IRI_{P28}-P56, and ^{CCND2}hiPSC-IR_{P28}-P35. Expressions of RYR2, MYH7, and ACTCT identify cardiomyocytes.

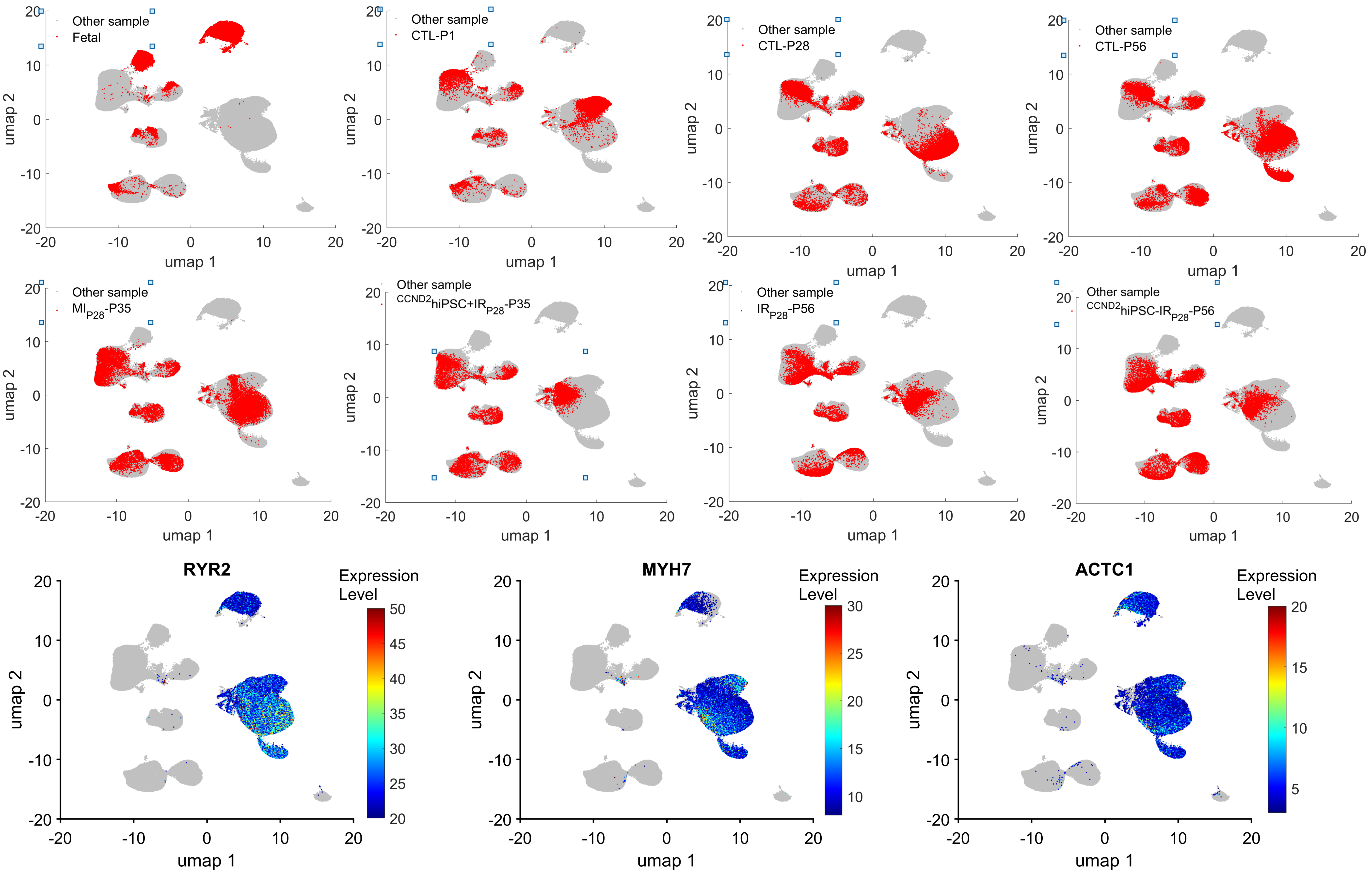

Supplemental Figure 2

UMAP plot of all cardiomyocytes after Autoencoder clustering, cell localization of the groups (Fetal, CTL-P1, CTL-P28, CTL-P56, MI_{P28}-P35, ^{CCND2}hiPSC-IR_{P28}-P35, IRI_{P28}-P56, and ^{CCND2}hiPSC-IR_{P28}-P35.

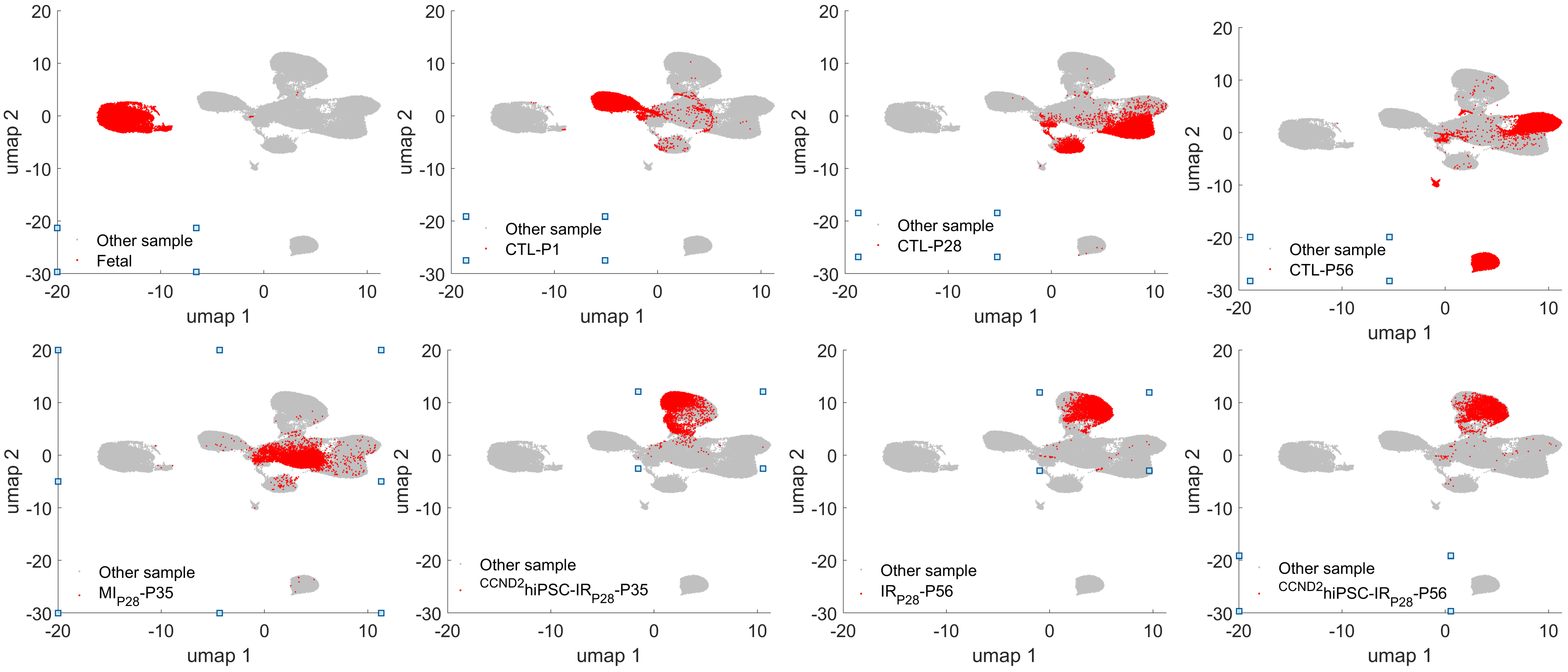

Supplemental Figure 3

Cluster analysis identified three non-cardiomyocyte cell-types in mouse hearts. Cluster analysis of scRNAseq data from mouse hearts was conducted via (A) AI Autoencoder, (B) Seurat, SC3, RaceID, and CIDR (using the same UMAP), (C) ScanPY, (D) scDHA, (E) ssCCEES, or (F) DCA; then, (Columns i-iii) expression of (i) the fibroblast marker Col1a1, (ii) the endothelial-cell marker *Pecam1*, (iii) the immune-cell marker *Ifi30*, and (iv) the smooth muscle cell marker *Tagln* was quantified across the corresponding UMAP and presented as a heat map.

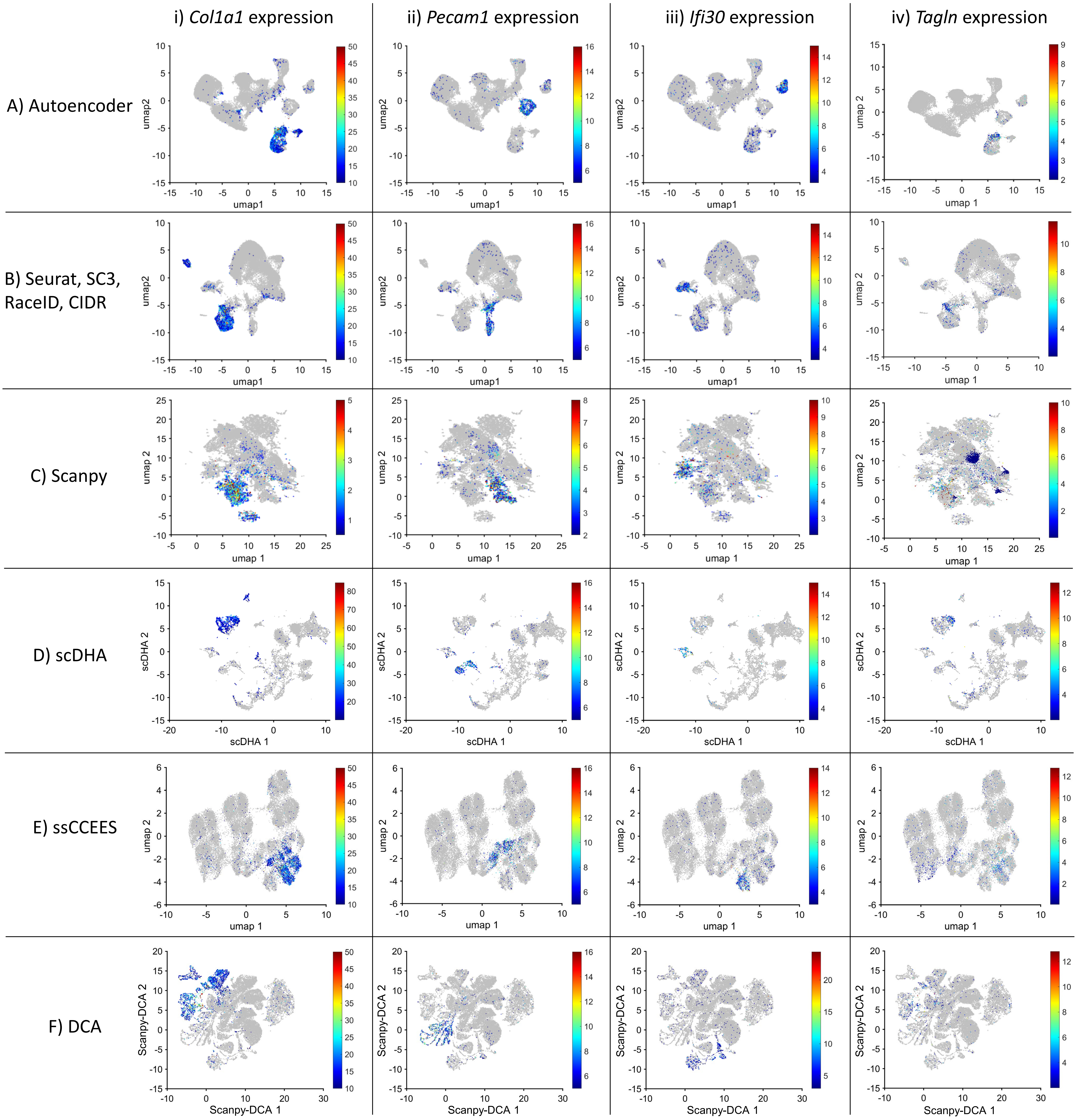

Supplemental Figure 4

Each cell-type specific cluster contained cells from all injury groups and time points. Cluster analysis of scRNAseq data from mouse hearts was conducted via (A) AI Autoencoder, (B) Seurat, SC3, RaceID, and CIDR, or (C) ScanPY and presented as a UMAP; then, (Rows) cardiomyocytes in hearts collected from each injury group and at each time point were displayed in red.

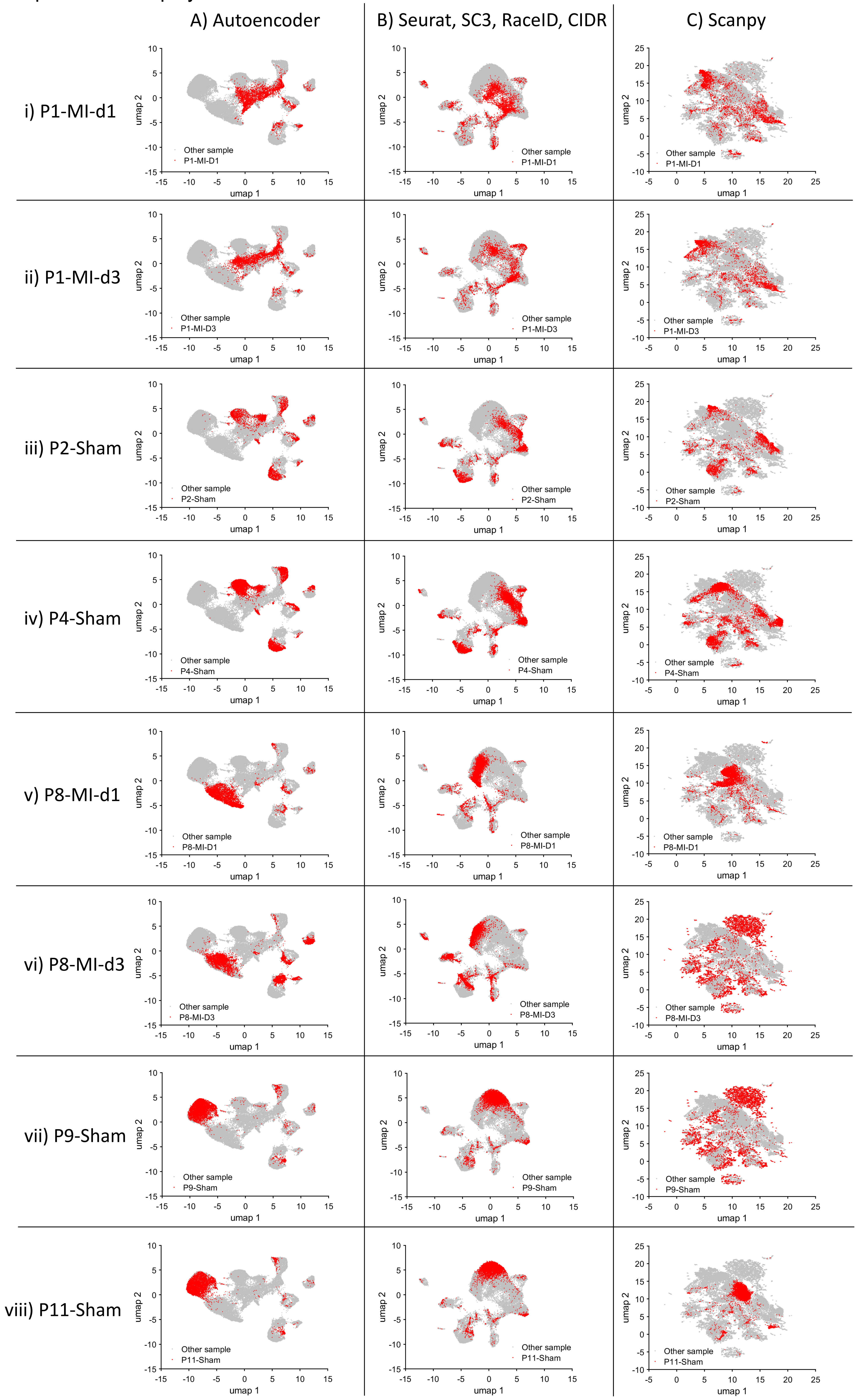

Supplemental Figure 5

Ventricular and atrial cardiomyocyte clusters were identified in the human cell atlas scRNAseq data using AI-based Autoencoder, Seurat, and ScanPY. The subfigures are organized in a grid structure. Each column (A-C) corresponds to a pipeline. Each row corresponds to a gene expression, in order: cardiomyocyte (overall) markers TNNT2 (i), TTN (ii), RYR2 (iii), ventricular cardiomyocyte markers MYH7 (iv), MYL2 (v), IRX3 (vi), and atrial cardiomyocyte marker HAMP (vii). The 2D landscape in this figure is identical to the corresponding landscape in Figure 6.

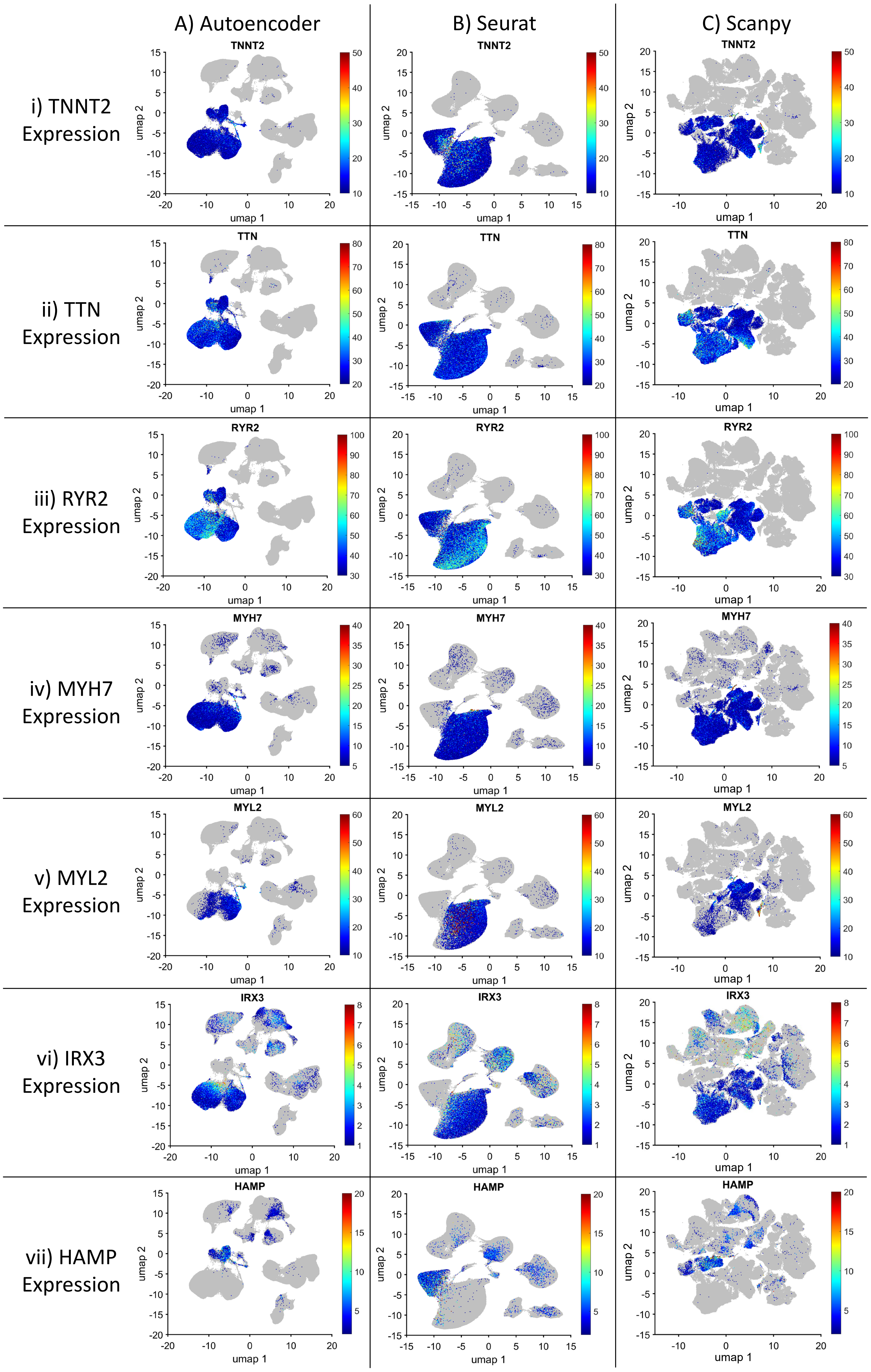

Supplemental Figure 6

Endothelial cells, pericytes, and smooth muscle cells were identified in the human cell atlas scRNAseq data using AI-based Autoencoder, Seurat, and ScanPY. The subfigures are organized in a grid structure. Each column (A-C) corresponds to a pipeline. Each row corresponds to a gene expression, in order: endothelial cell markers CDH5 (i), PECAM1 (ii), VWF (iii), pericyte markers ABCC9 (iv), KCNJ8 (v), smooth muscle cell marker TAGLN (vi) and ACTA2 (vii). The 2D landscape in this figure is identical to the corresponding landscape in Figure 6.

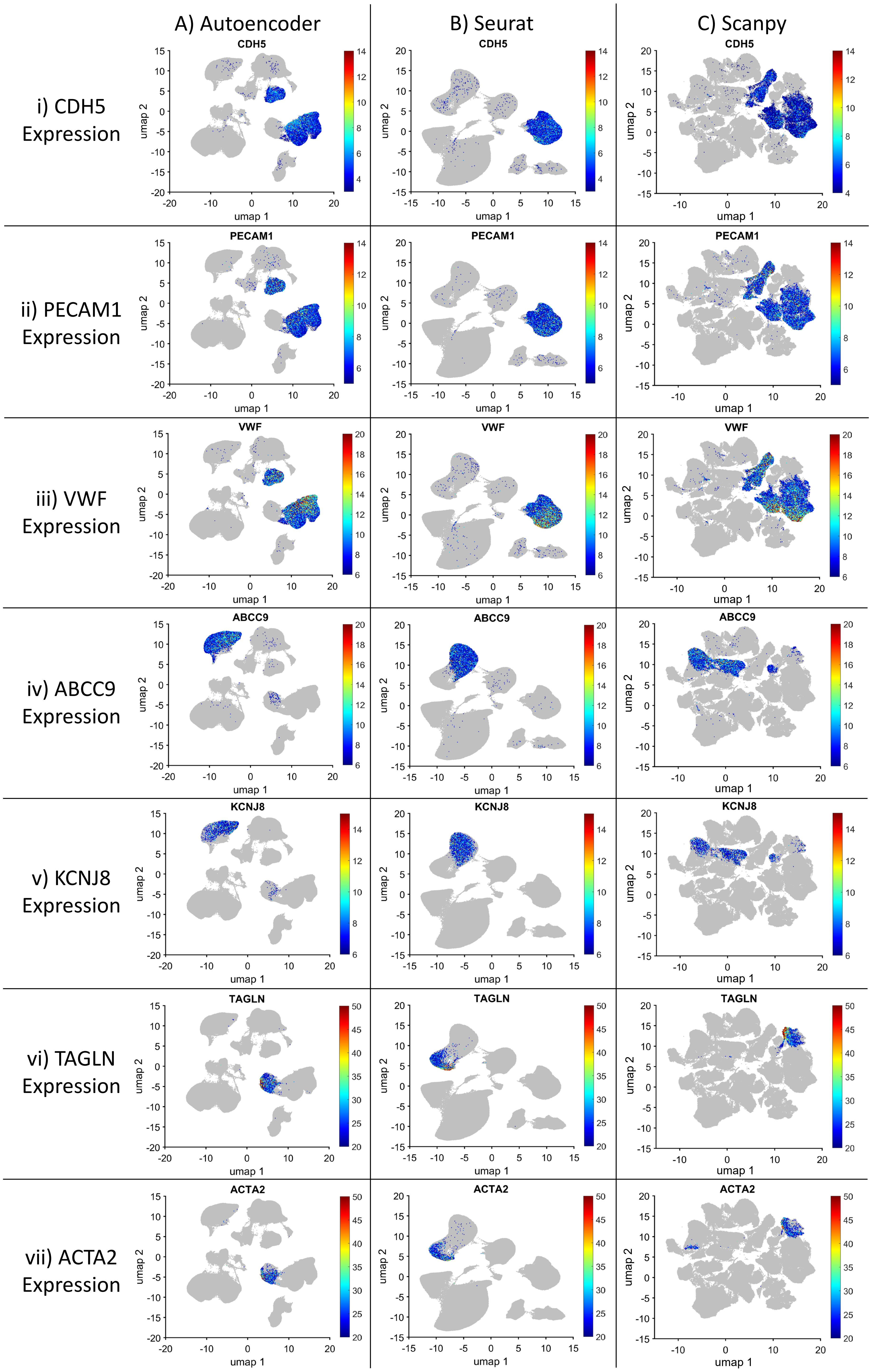

Supplemental Figure 7

Monocyte-macrophages and lymphocytes were identified in the human cell atlas scRNAseq data using AI-based Autoencoder, Seurat, and ScanPY. The subfigures are organized in a grid structure. Each column (A-C) corresponds to a pipeline. Each row corresponds to a gene expression, in order: monocyte-macrophage markers CD163 (i), LYVE1 (ii), lymphocyte markers CD3E (iii), CD3G (iv), and CD8A (v). The 2D landscape in this figure is identical to the corresponding landscape in Figure 6.

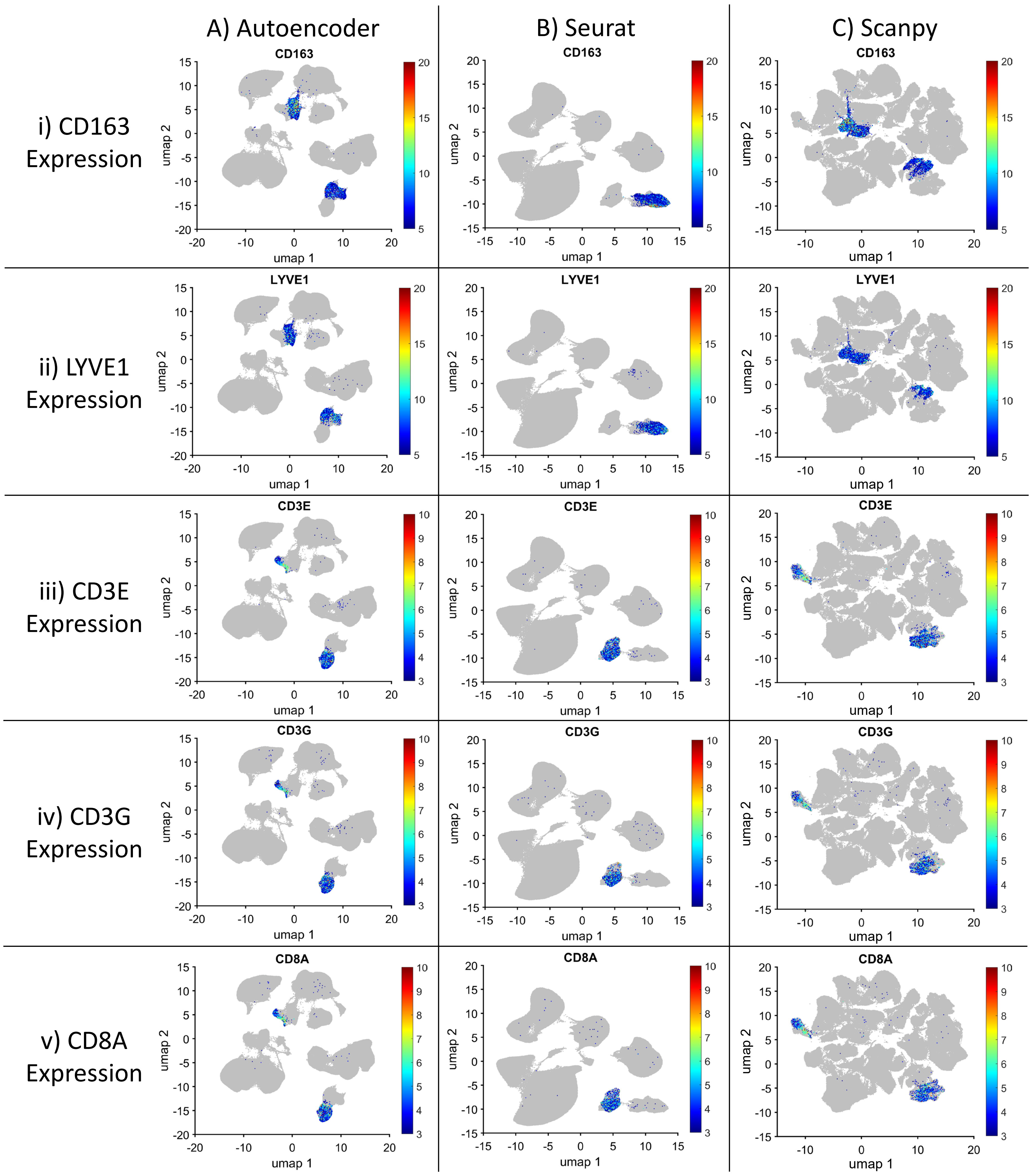

Supplemental Figure 8

Fibroblasts and Glial cells were identified in the human cell atlas scRNAseq data using AI-based Autoencoder, Seurat, and ScanPY. The subfigures are organized in a grid structure. Each column (A-C) corresponds to a pipeline. Each row corresponds to a gene expression, in order: fibroblast markers DCN (i), GSN (ii), PDGFRA (iii), glial cell markers NRXN1 (iv), NRXN3 (v), and KCNMB4 (vi). The 2D landscape in this figure is identical to the corresponding landscape in Figure 6.

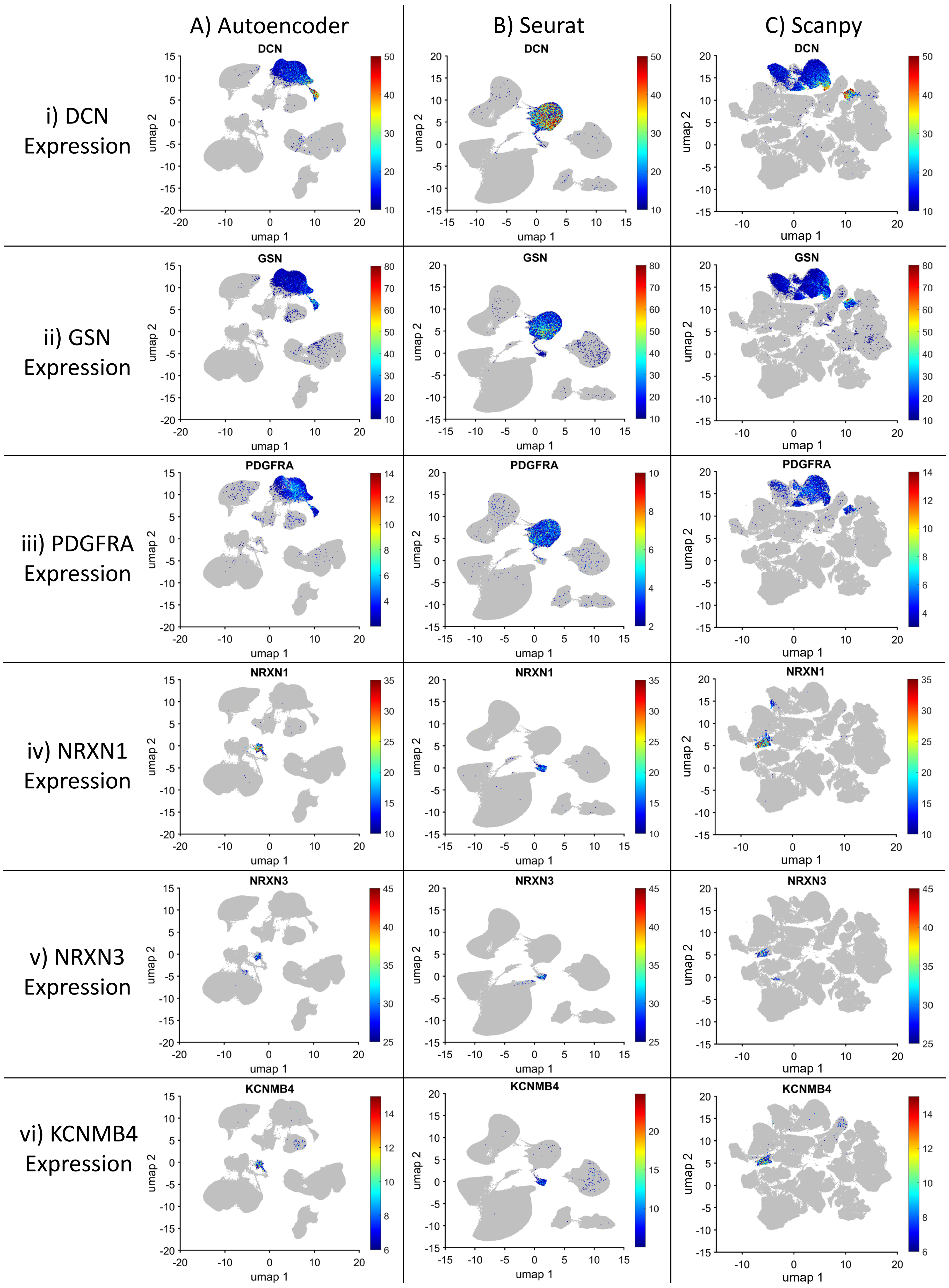

Supplemental Figure 9

Using the AI-based Autoencoder embedding, different clustering algorithms result in consistent cluster cell types. After being embedded into just 10 dimension by the Autoencoder, the pig scRNAseq data was clustered by three clustering algorithms, then the cluster results were visualized on the same UMAP coordinate and the same cell type identification method to[37]. A) K-mean clustering result. B) Louvain clustering result. C) Density-based clustering result.

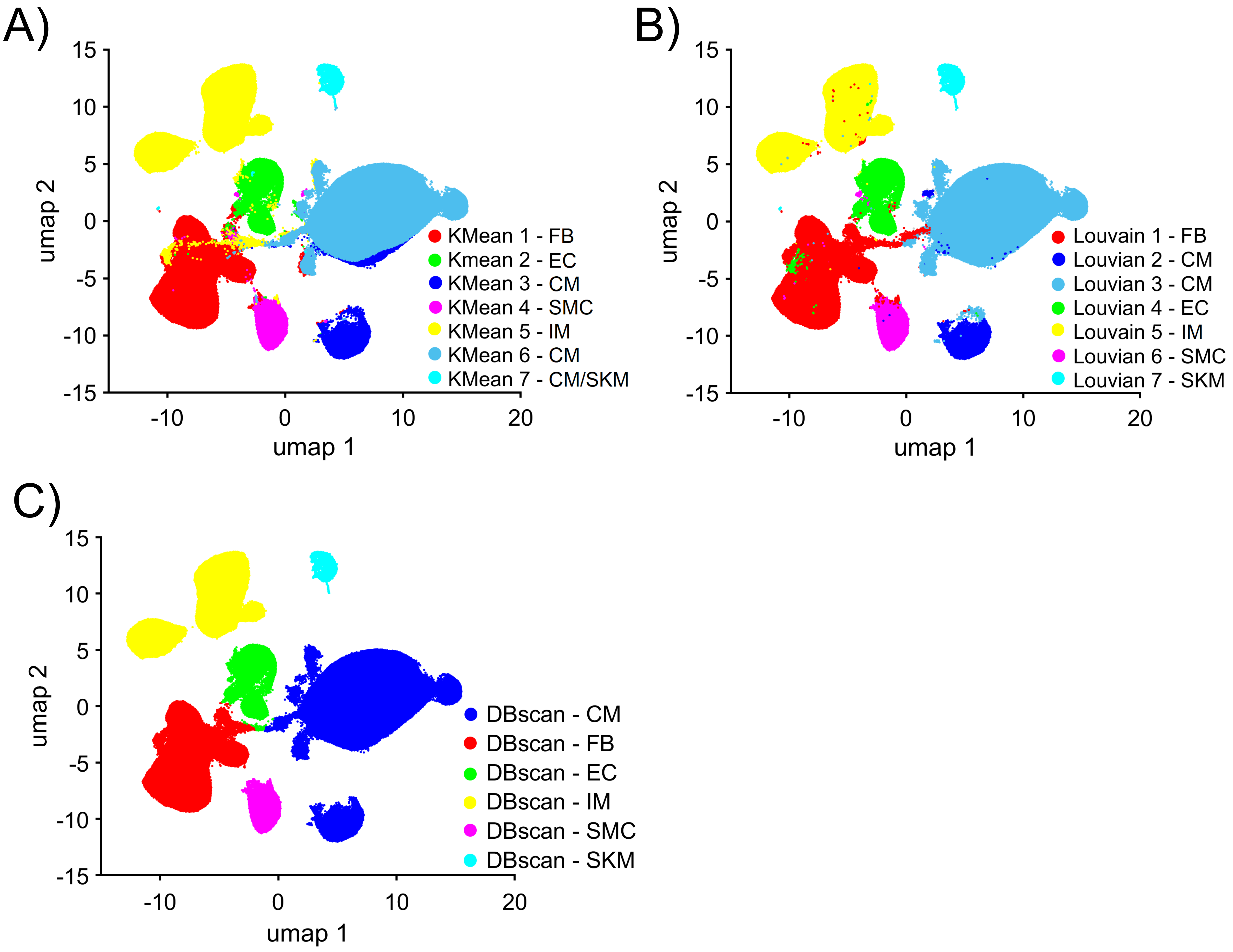

Supplemental Figure 10

Analyzing pathways and biological process upregulated in ^{CCND2}hiPSC-IR$_{P28}$-P35 cardiomyocytes, compared to MI$_{P28}$-P35 cardiomyocytes. Methods that do not calculate enrichment for each cardiomyocyte include: A) Gene ontology results, produced by DAVID (https://david.ncifcrf.gov) with Seurat-Ranksum result. B) Gene ontology results, produced by DAVID (https://david.ncifcrf.gov) with Seurat-MAST result. Methods that calculate enrichment for each cardiomyocyte include AI-based sparse model and ssGSEA; therefore, violin plots, which summarize the enrichment scores for all cells, were examined among all groups. From C) to L), the left subpanel is the spare model result, and the right subpanel is the ssGSEA result. C) Cell cycle G1 to DNA synthesis phase (G1S). D) Cell cycle DNA synthesis phase (S). E) Cell cycle G2 to Mitosis checkpoint phase (G2M). F) Cytokinesis phase. G) MAPK signaling. H) HIPPO signaling. I) cAMP signaling. J) JAK-STAT signaling. K) RAS signaling. L) TGFβ signaling.

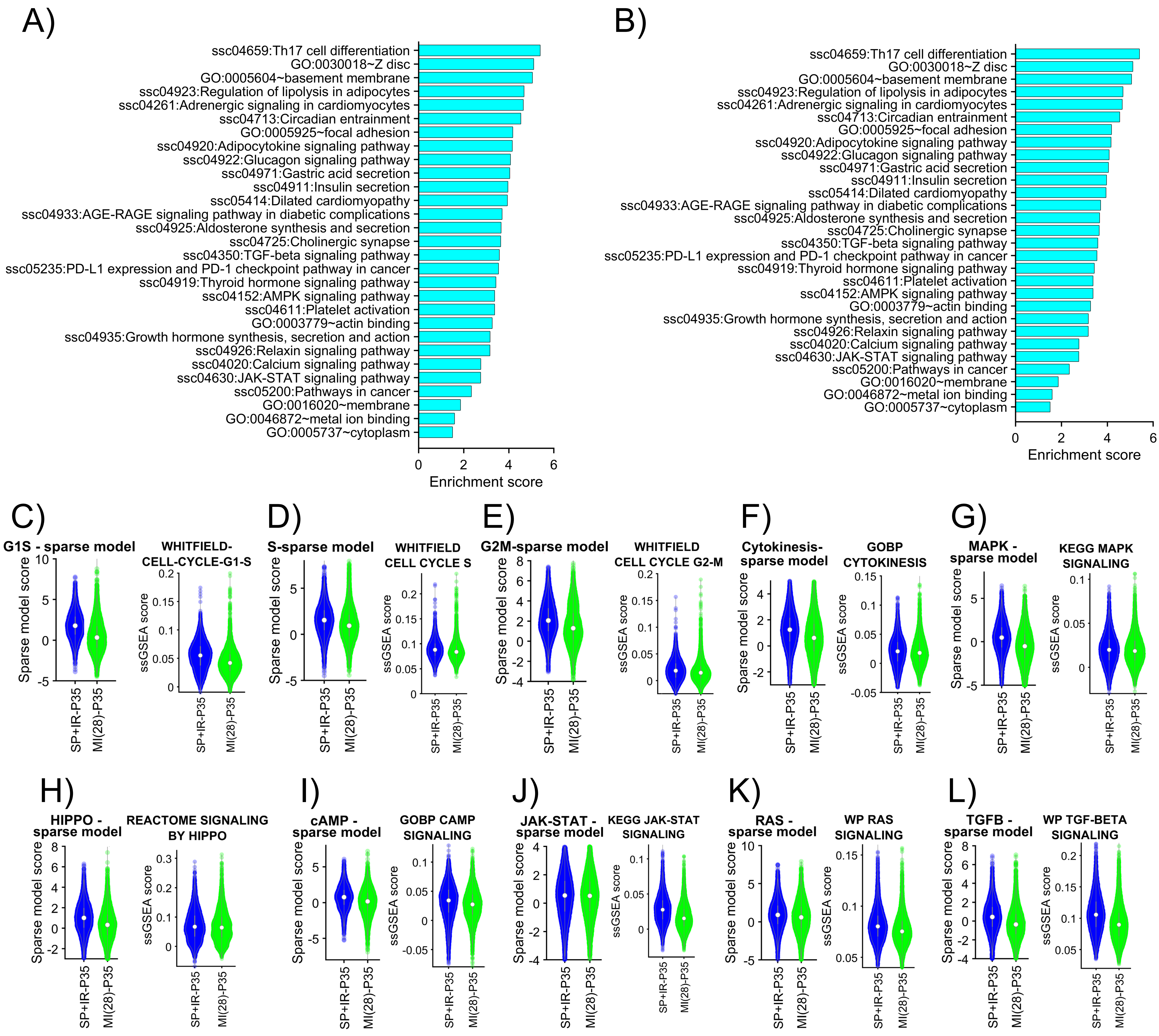

Supplemental Figure 11

Separation between Human and Pig cells in scRNAseq data. Pig cells: 6095 pig heart cells selected from our lab's data[18]. Human cell: 4737 cells selected from iPSC data[19]. A) The % of SC transcripts mapped to the Human (GRch38) and Pig (Sscrofa10.2) genomes, produced by Cell Ranger pipeline; this result shows that the current 'published' human and 'draft' pig genome is sufficient to distinguish between human and pig cells. B) Combined' human+pig cells on human+pig genome' UMAP plot of the SC data following Cell Ranger. C) Histograms of ratio between the number of pig-genome-mapped transcripts and the number of human-genome-mapped transcripts in each cell; the cyan histogram represents the human cell; the red histogram represents the pig cell. D) UMAP plot of the cells, where the ratio in figure C is demonstrated.

A)

Reference Genome	SC Data: Human	SC Data: Pig
Human	**95.6%**	3.6%
Pig	28.2%	**92.6%**

B)

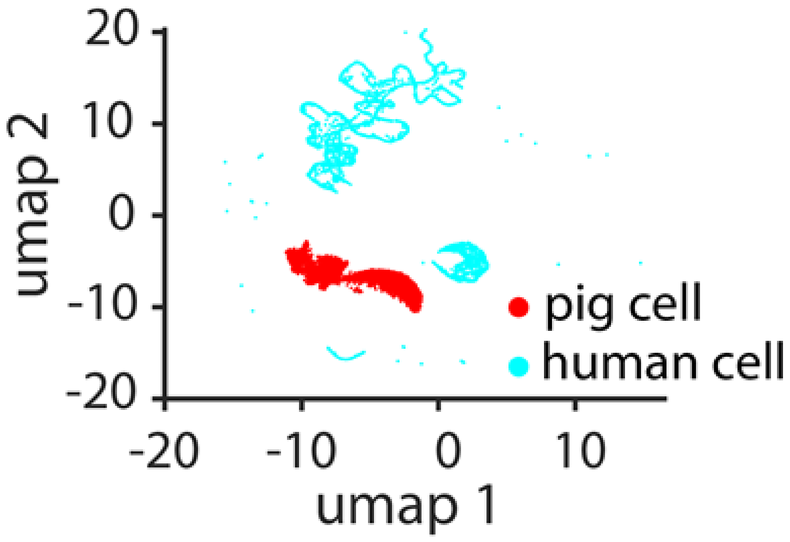

C)

D)

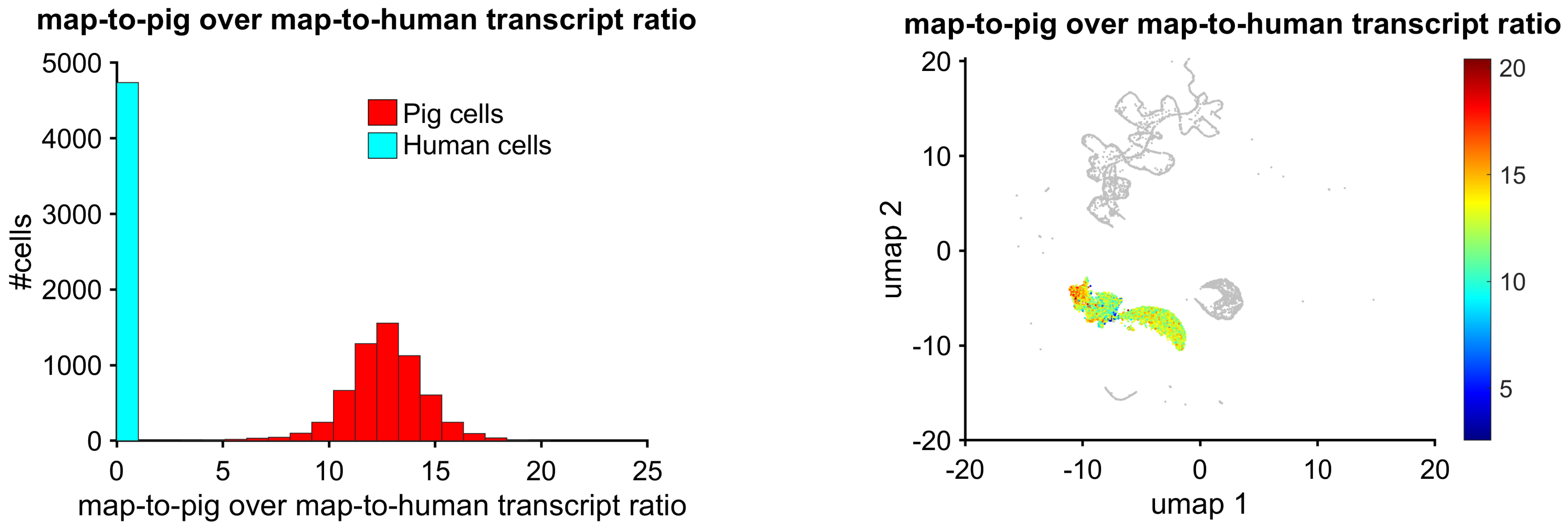

Supplemental Table 1. Curated cytokinetic gene

Gene	#Cytokineis-specific term	#Other term	Ratio
Cntrob	12	4	3
Aurkb	48	24	2
Klhdc8b	8	8	1
Anln	10	11	0.909091
Alkbh4	10	11	0.909091
Mitd1	6	8	0.75
Cep55	9	13	0.692308
Racgap1	17	29	0.586207
Ect2	15	28	0.535714
Kif20b	17	34	0.5
Nup62	21	45	0.466667
Bin3	5	12	0.416667
Chmp7	12	30	0.4
Chmp4c	12	32	0.375
Chmp5	11	34	0.323529
Chmp1b	11	36	0.305556
Chmp4b	14	49	0.285714
Chmp6	9	32	0.28125
Kif20a	5	19	0.263158
Chmp2a	11	43	0.255814
Vps4b	13	51	0.254902
Spast	11	47	0.234043
Anxa11	5	22	0.227273
Chmp1a	11	51	0.215686
Pdcd6ip	8	38	0.210526
Snx18	5	26	0.192308
Snx33	5	26	0.192308
Rtkn	3	16	0.1875
Chmp2b	7	44	0.159091
Vps4a	10	64	0.15625
Spire2	4	28	0.142857
Snx9	6	45	0.133333
Spire1	5	39	0.128205
Stx2	3	41	0.073171
Rhoa	6	149	0.040268
Arf1	2	57	0.035088
Myh9	3	131	0.022901
Plec	2	112	0.017857
